# The interplay between fluorescence and phosphorescence with luminescent gold(i) and gold(iii) complexes bearing heterocyclic arylacetylide ligands†
†Electronic supplementary information (ESI) available: Experimental details of synthesis, characterization, photophysical data and additional computational details. CCDC 1499919–1499921. For ESI and crystallographic data in CIF or other electronic format see DOI: 10.1039/c6sc03775e



**DOI:** 10.1039/c6sc03775e

**Published:** 2016-12-05

**Authors:** Kaai Tung Chan, Glenna So Ming Tong, Wai-Pong To, Chen Yang, Lili Du, David Lee Phillips, Chi-Ming Che

**Affiliations:** a State Key Laboratory of Synthetic Chemistry , Institute of Molecular Functional Materials , Department of Chemistry , The University of Hong Kong , Pokfulam Road , Hong Kong SAR , China . Email: tongsm@hku.hk ; Email: cmche@hku.hk; b Department of Chemistry , The University of Hong Kong , Hong Kong , China; c Department of Chemistry , HKU Shenzhen Institute of Research and Innovation , Shenzhen 518053 , China

## Abstract

The photophysical properties of a series of gold(i) [LAu(C≡CR)] (L = PCy_3_ (**1a–4a**), RNC (**5a**), NHC (**6a**)) and gold(iii) complexes [Au(C^N^C)(C≡CR)] (**1b–4b**) bearing heterocyclic arylacetylide ligands with narrow band-gap are compared. The luminescence of both series are derived from an intraligand transition localized on the arylacetylide ligand (ππ*(C≡CR)) but **1a–3a** displayed prompt fluorescence (*τ*_PF_ = 2.7–12.0 ns) while **1b–3b** showed mainly phosphorescence (*τ*_Ph_ = 104–205 μs). The experimentally determined intersystem crossing (ISC) rate constants (*k*_ISC_) are on the order of 10^6^ to 10^8^ s^–1^ for the gold(i) series (**1a–3a**) but 10^10^ to 10^11^ s^–1^ for the gold(iii) analogues (**1b–3b**). DFT/TDDFT calculations have been performed to help understand the difference in the *k*_ISC_ between the two series of complexes. Owing to the different oxidation states of the gold ion, the Au(i) complexes have linear coordination geometry while the Au(iii) complexes are square planar. It was found from DFT/TDDFT calculations that due to this difference in coordination geometries, the energy gap between the singlet and triplet excited states (Δ*E*_ST_) with effective spin–orbit coupling (SOC) for Au(i) systems is much larger than that for the Au(iii) counterparts, thus resulting in the poor ISC efficiency for the former. Time-resolved spectroscopies revealed a minor contribution (<2.9%) of a long-lived delayed fluorescence (DF) (*τ*_DF_ = 4.6–12.5 μs) to the total fluorescence in **1a–3a**. Attempts have been made to elucidate the mechanism for the origins of the DF: the dependence of the DF intensity with the power of excitation light reveals that triplet–triplet annihilation (TTA) is the most probable mechanism for the DF of **1a** while germinate electron–hole pair (GP) recombination accounts for the DF of **2a** in 77 K glassy solution (MeOH/EtOH = 4 : 1). Both **4a** and **4b** contain a BODIPY moiety at the acetylide ligand and display only ^1^IL(ππ*) fluorescence with negligible phosphorescence being observed. Computational analyses attributed this observation to the lack of low-lying triplet excited states that could have effective SOC with the S_1_ excited state.

## Abstract 2

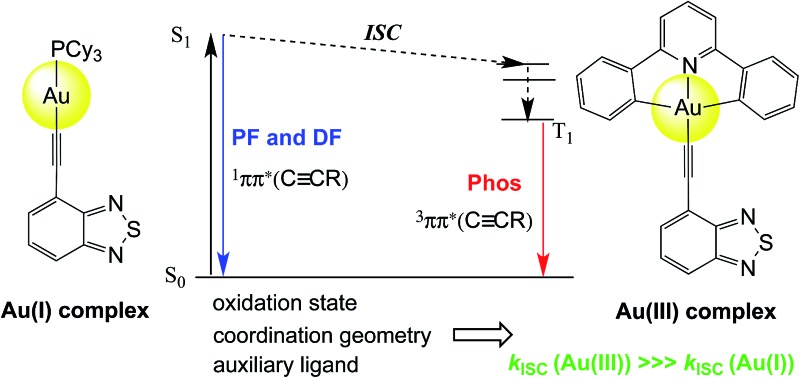
The rates of intersystem crossing of two families of gold complexes are significantly influenced by the oxidation state of the metal ion, which dictates the coordination geometries.

## Introduction

Phosphorescence is a distinctive photophysical property of transition-metal complexes, which has widespread applications in diverse areas. As it is derived from the ‘forbidden’ radiative relaxation of a triplet-excited state to the singlet ground state, it is featured by long emission lifetime (in μs) and reduced emission energy compared to fluorescence commonly encountered in organic luminophores. Electronic transitions associated with a change of spin are prohibited by the spin-selection rule. However, transition-metal ions that have high atomic number and hence, large spin–orbit coupling constant (*ξ*), usually lead to efficient spin–orbit coupling (SOC) that relaxes the spin selection rule. Fast intersystem crossing (ISC) in the sub-picosecond to picosecond time regime^1^ leads to rapid depletion of a singlet excited state to a triplet excited state instead of fluorescence as fluorescence radiative lifetime is typically in the nanosecond range. Thus, in transition-metal complexes, phosphorescence normally prevails in their luminescence spectra.

**Fig. 1:**
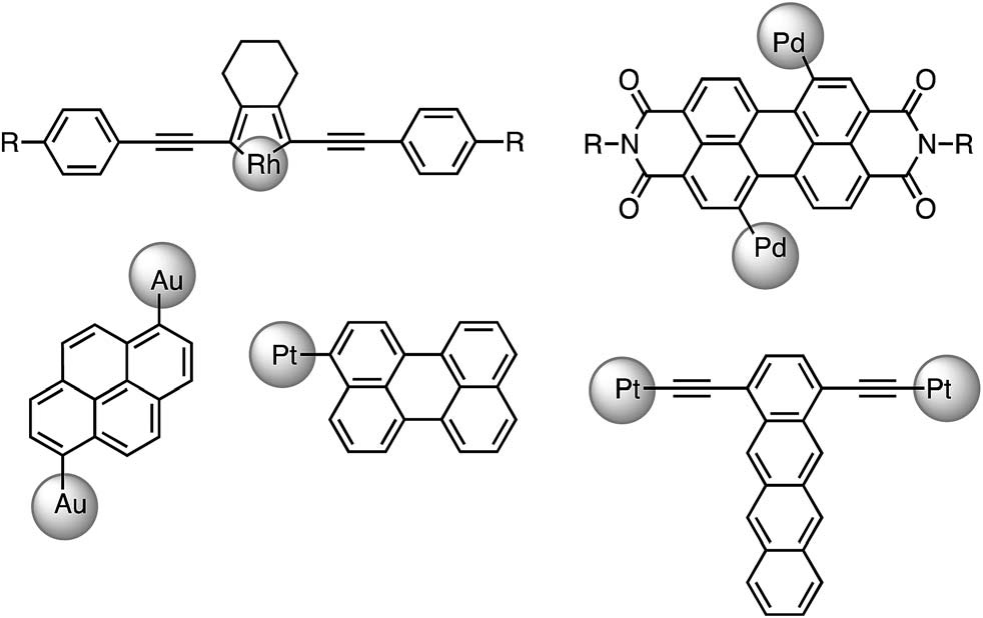
Selected examples of transition-metal complexes that display dominant fluorescence instead of phosphorescence. Auxiliary ligands coordinated to the metal ions are omitted.

However, in recent years, there are an increasing number of reports on transition-metal complexes which display slow ISC rate with lifetimes ranging from hundreds of ps to ns. For instance, 2,5-bis(arylethynyl)rhodacyclopentadiene complexes (*ξ*_Rh_ = 1260 cm^–1^)^2^ were reported to display exclusively prompt fluorescence with high emission quantum yields of 0.3–0.7 and lifetimes of 1–3 ns, corresponding to ISC rate constants of ∼10^8^ s^–1^.^3^ In addition, transition-metal complexes containing fused aromatic systems such as perylene, perylene diimide, pyrene and tetracene also show ligand-dominated fluorescence (see Fig. 1).^4^ Hence, it has become clear that the presence of heavy elements does not guarantee fast ISC rate; the molecular structure and the nature of the ligands may play more critical roles in determining the ISC rate.

Luminescent Au(i) complexes are well documented to display rich photophysical properties. Although Au(i) complexes generally display phosphorescence owing to the large SOC constant of Au(i) ion (*ξ*_Au_ ∼ 5100 cm^–1^),^2^ ligand-centered fluorescence has also been reported in a number of gold(i) complexes. For example, as revealed by the luminescence of [TEE(AuPCy_3_)_4_] and [TEB(AuPCy_3_)_3_] (TEE = tetraethynylethene; TEB = 1,3,5-triethynylbenzene), subtle changes in the electronic structure of the bridging alkynyl ligand leads to intense phosphorescence (*Φ*_em_ = 0.46, *τ* = 285 μs) in the latter but solely fluorescence (*Φ*_em_ = 0.22, *τ* < 0.05 μs) in the former.^5^ In both cases, the luminescence originates from the ligand-centered transition mainly localized on the bridging alkynyl ligands. Che and co-workers also reported a series of Au(i)-conjugated acetylides, [(Cy_3_P)Au(C≡C–C_6_H_4_)_*n*–1_(C≡CPh)] (*n* ≥ 2), which display dual fluorescence (prompt and delayed) and phosphorescence.^6^ Both the *Φ*_em_ and ratio of fluorescence *versus* phosphorescence were found to depend on the conjugation length (number of repeating units *n*) and the substitution pattern of arylacetylide ligands.

**Chart 1:**
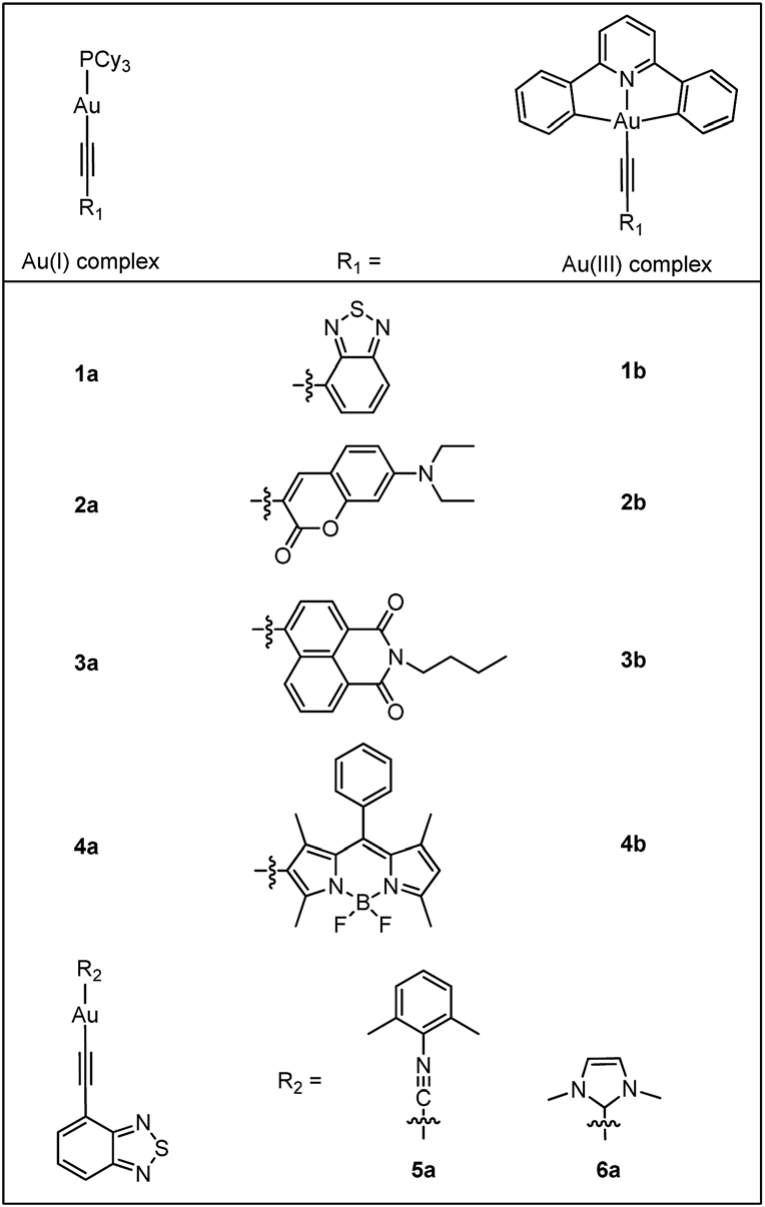
Au(i) and Au(iii) acetylide complexes studied in this work.

As a continuous effort to elucidate the ligand effects on the photophysics of luminescent gold complexes, heterocyclic arylacetylide ligands containing narrow band-gap moieties (benzothiadiazole (L1), coumarin (L2), naphthalimide (L3) and boron-dipyrromethene (referred to as Bodipy) (L4); Chart 1) were chosen in this study. A series of Au(i) heterocyclic arylacetylide complexes, **1a–4a**, were synthesized. Tricyclohexylphosphine (PCy_3_) was used as the auxiliary ligand in these complexes because (1) it is optically transparent at wavelength >250 nm so that it is not involved in the emissive excited states in the UV-visible spectral region and (2) its steric bulkiness would prevent the gold ions from coming into close contact that could lead to low-lying excited states originated from metal–metal and π–π interactions. It is worth mentioning that several recently reported Au(i) alkynyl complexes bearing similar benzothiadiazole,^7^ coumarin^8^ and naphthalimide^9^ derivatives also show similar luminescence properties as our Au(i) complexes.^10,11^ The effects of auxiliary ligands on the photophysical properties of Au(i) complexes were also studied by comparing **1a** with two derivatives containing 2,6-dimethylphenyl isocyanide (RNC, **5a**) and 1,3-dimethylimidazol-2-ylidene (NHC, **6a**) instead of the phosphine auxiliary ligand, respectively.

The effects of the oxidation state of the metal ion on the photophysical behaviours of transition-metal complexes are relatively unexplored. Herein, an analogous series of Au(iii)-acetylides supported by the cyclometalated [C^N^C] ligand (**1b–4b**; HC^N^CH = 2,6-diphenylpyridine) were also prepared and their photophysical properties were compared with those of the Au(i) counterparts. The photophysical properties of both Au(i) and Au(iii) complexes were investigated by steady-state and time-resolved spectroscopic measurements. DFT/TDDFT calculations were performed on the pairs (**1a**, **1b**) and (**4a**, **4b**) in order to understand the origin of the dramatic difference in ISC efficiencies between these Au(i) and Au(iii) complexes.

## Results

### Synthesis and characterization

The gold(i) alkynyl complexes **1a–6a** were synthesized in 53–79% yields following the protocol of base deprotonation (NaOMe) of terminal alkynes and substitution of chloride ion of the corresponding Au(i) precursors.^5a,6a,b,12^ As these complexes were observed to show signs of decomposition on SiO_2_ column, column chromatography was not used for their purification. Analytically pure **1a–6a** were obtained by recrystallization from CH_2_Cl_2_/hexane mixtures. The Au(iii) complexes **1b–4b** were synthesized by copper-catalyzed Sonogashira coupling between terminal alkynes and [Au(C^N^C)Cl] using deoxygenated CH_2_Cl_2_ as the solvent, with NEt_3_ added to initiate the deprotonation of alkynes.^13^ These complexes were purified by chromatography on SiO_2_ column using dichloromethane and hexane as eluent. The yields were 51–84%.

All complexes have been characterized by ^1^H and ^13^C NMR, mass spectrometry (FAB+) and elemental analyses. Ligands L1–L4 were characterized by ^1^H NMR and MS-EI. The complexes are stable in the solid state and in solution under ambient conditions. Complexes **1a–6a** are highly soluble in CH_2_Cl_2_ and THF but are less soluble in alcoholic solvents such as MeOH. Complexes **1b–4b** have lower solubility compared with their Au(i) counterparts. All of these gold complexes appear as yellow or orange solids except for **4a** and **4b** that are purplish red. The ^31^P signals of **1a–4a** occur at *ca. δ* 56.3 as a singlet, characteristic of the ^31^P signals of the Au–PCy_3_ moiety that usually appear in the range of *δ* 56.0–58.0.^6a,b,12^ In the ^13^C NMR spectra, two doublets are observed at *ca. δ* 131.2–146.2 (^2^ *J*_CP_ ≈ 130 Hz) and 94.6–98.4 (^3^ *J*_CP_ ≈ 24 Hz) which can be assigned to the α and β-acetylenic carbons.^6a,b,12b^ In **6a**, the carbene carbon ligated to gold occurs at *δ* 187.7.^14^

### X-Ray crystallography

**Fig. 2:**
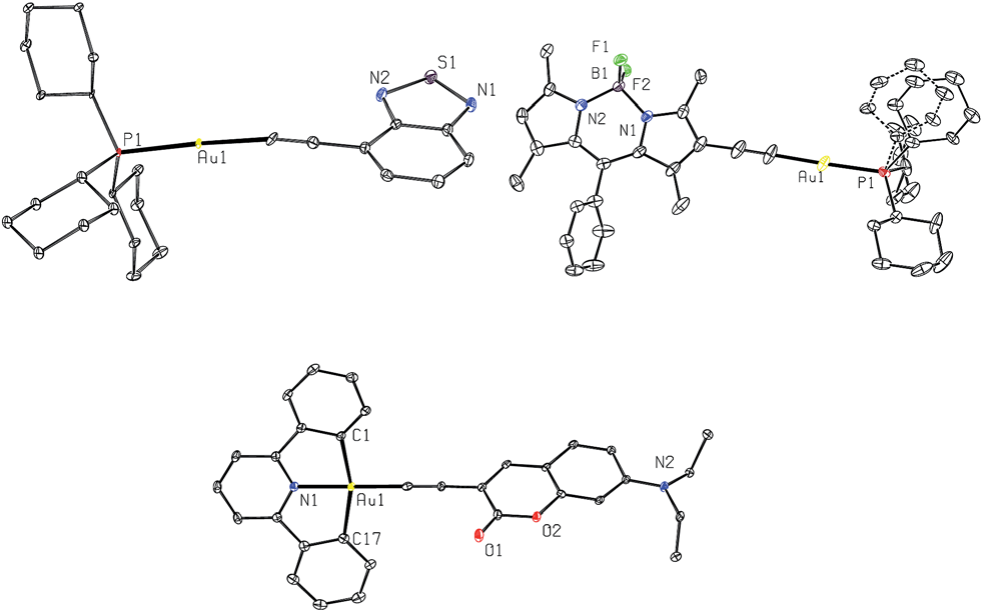
Perspective drawings of the crystal structures of **1a** (top left), **4a** (top right) and **2b** (bottom) with the thermal ellipsoids shown at 30% probability level. Hydrogen atoms have been omitted for clarity.

Crystals of **1a**, **4a** and **2b** were obtained by layering hexane over concentrated CH_2_Cl_2_ solutions. Their crystal data and selected bond lengths and angles are given in ESI.† Fig. 2 shows the structures of **1a** and **4a** (top panel). The P1–Au1–C(acetylide) angles of **1a** and **4a** are 175.0(13) and 178.1(2)° and Au1–C≡C angles are 170.9(4) and 177.6(5)°, respectively, revealing slight deviation from linear coordination geometry. The Au1–C(acetylide) distances of 2.049(4) and 2.001(5) Å and C≡C distances of 1.146(7) and 1.191(8) Å for **1a** and **4a**, respectively, are comparable with those of other reported gold(i) acetylide complexes.^6a,b,12a,b^ The crystal packing diagrams of **1a** and **4a** are shown in ESI (Fig. S1†). In both cases, there are no short intermolecular contacts; the closest Au···Au distances are 5.9715(5) and 5.2726(4) Å for **1a** and **4a**, respectively.

The crystal structure of **2b** (Fig. 2, bottom) shows a slightly distorted square-planar geometry with C1–Au1–C17 angle of 162.44(14)°. The Au1–C(acetylide) and C≡C distances are 1.969(4) and 1.197(5) Å, respectively. These parameters are similar to those found in related cyclometalated Au(iii) arylacetylide complexes.^13^ The torsional angle between the Au(C^N^C) and arylacetylide planes is approximately 72.6°. This non-planarity gives rise to negligible π–π stacking between molecules as shown in Fig. S2 in ESI.†

### Electrochemical properties

The electrochemical properties of selected complexes, **1a–4a** and **1b–4b**, were investigated by cyclic voltammetry. The electrochemical data are summarized in Table 1. The cyclic voltammograms of the Au(i) complexes and their Au(iii) counterparts are shown in Fig. S3, ESI.† Except for **1a** and **2a**, both classes of complexes display both irreversible oxidation (*E*_pa_ = +0.6 to +1.4 V) and quasi-reversible/irreversible reduction waves (*E*_pc_ = –1.5 to –1.8 V) attributed to the redox process localized on the arylacetylides. For the pairs [**2a**, **2b**] and [**4a**, **4b**], the *E*_pa_ occur at relatively low potential of *ca.* +0.6 and +0.7 V, respectively, suggesting higher HOMO level of the conjugated coumarin and Bodipy than the other heterocyclic moieties. For the Au(iii) complexes, other than the redox reactions occurring at the arylacetylide ligands at potentials similar to the Au(i) counterparts, there are also irreversible reduction waves at *ca.* –1.9 to –2.0 V attributable to reduction of the [C^N^C] ligands.

**Table 1:** Electrochemical data of **1a–4a** and **1b–4b** ^*a*^

Complex	*E*_pc_ ^*b*^/V	*E*_pa_ ^*c*^/V
**1a**	–1.79^*d*^	—
**2a**	—	0.61, 0.95
**3a**	–1.61^*d*^	1.44
**4a**	–1.50^*d*^	0.71, 1.14
**1b**	–1.82, –2.03	1.25
**2b**	–1.89	0.59, 1.02
**3b**	–1.59, –1.95	1.42
**4b**	–1.49^*d*^, –2.02	0.74

^*a*^Values determined in CH_2_Cl_2_ (Cp_2_Fe^+/0^ occurs at *E*_1/2_ = +0.15–0.16 V) at 298 K; values reported *versus* Ag/AgNO_3_ reference electrode; electrolyte: 0.1 M *n*Bu_4_NPF_6_; scan rate = 100 mV s^–1^.

^*b*^Cathodic peak potential (*E*_pc_) of irreversible wave.

^*c*^Anodic peak potential of (*E*_pa_) irreversible wave.

^*d*^ *E*_1/2_ = *E*_pa_ + *E*_pc_ of quasi-reversible wave.

### UV-vis absorption spectroscopy

All photophysical data of the gold complexes and the free ligands L1–L4 are listed in Table 2.

**Table 2:** Photophysical data of **1a–6a**, **1b–4b** and L1–L4^*a*^

	UV/Vis absorption, *λ*_max_/nm (10^3^ *ε*/M^–1^ cm^–1^)	Emission						
		Medium	*λ*_F_/nm	*λ*_Ph_/nm	*τ*_PF_ ^*b*^/ns	*τ*_DF_ ^*c*^/μs	*τ*_phos_/μs	*Φ*_em_ ^*d*^
**1a**	265 (14.1), 275 (14.6), 305 (9.3), 311 (9.8), 319 (12.4), 379 (6.9)	CH_2_Cl_2_ 298 K	467	—	12.0	11.9	—	0.91
		Glassy 77 K	442	630, 688^*e*^		n.d.^*f*^	109^*e*^	
		Solid 298 K	504	—		5.3	—	
		Solid 77 K	492	—		11.6	—	

**2a**	271 (13.9), 316 (3.2), 331 (3.2), 410 (40.0), 423 (3.7, br)	CH_2_Cl_2_ 298 K	466	596, 652^*e*^	2.7	4.6	n.d.^*f*^	0.70
						11.8^*g*^	13.6^*g*^	
		Glassy 77 K	460, 481	596, 653^*e*^		243^*e*^	203^*e*^	
		Solid 298 K	405 (weak), 480 (sh), 515 (max)	598, 652 (sh)^*e*^		15.0^*e*^	24.6^*e*^	
		Solid 77 K	409 (weak), 487 (max), 517 (sh)	597, 654(sh)^*e*^		9.4^*e*^	12.0^*e*^	

**3a**	283 (12.2), 333 (5.6), 350 (11.3), 380 (23.8), 397 (25.5)	CH_2_Cl_2_ 298 K	441	613, 670^*e*^	2.8	2.6, 12.5	n.d.^*f*^	0.78
						31.5^*g*^	61.9^*g*^	
		Glassy 77 K	418, 439, 462 (sh)	569, 609, 666^*e*^		n.d.^*f*^	530^*e*^	
		Solid 298 K	403 (sh), 503	610, 668^*e*^		29.1	64.9^*e*^	
		Solid 77 K	404 (sh), 504	627, 680^*e*^		35.1	92.3^*e*^	

**4a**	280 (13.5), 325 (4.5), 412 (10.1), 553 (40.2)	CH_2_Cl_2_ 298 K	593	—	0.8	—	—	0.04

**5a**	266 (18.6), 279 (15.8), 294 (12.5), 305 (11.2), 311 (10.1), 319 (12.7), 371 (7.1)	CH_2_Cl_2_ 298 K	456	—	11.0	6.8	—	0.90

**6a**	259 (18.3), 280 (17.5), 298 (7.8), 305 (10.7), 311 (11.4), 319 (14.2), 383 (8.3)	CH_2_Cl_2_ 298 K	476	—	14.0	7.4	—	0.84

**1b**	283 (20.4), 310 (24.1), 318 (26.8), 369 (13.0), 381 (13.8)	CH_2_Cl_2_ 298 K	461^*h*^	630, 671 (sh)	13.8^*h*^	—	104	0.003^*i*^
		Glassy 77 K		610, 668			n.d.^*f*^	

**2b**	312 (13.9), 406 (35.9, br), 432 (40.7)	CH_2_Cl_2_ 298 K	473^*h*^	592, 642 (sh)	9.0^*h*^	—	124	0.01^*i*^
		Glassy 77 K		585, 605, 643			1200	
		Solid 298 K		530, 601, 660			1.4	

**3b**	312 (12.7), 325 (11.9), 379 (26.6), 395 (29.5)	CH_2_Cl_2_ 298 K	459^*h*^	603, 659 (sh)	5.2^*h*^	—	205	0.04^*i*^
		Glassy 77 K		598, 614 (sh), 652			2200	

**4b**	312 (12.1), 320 (12.0), 366 (7.6), 384 (9.6), 401 (10.7), 515 (24.0, br), 546 (41.7)	CH_2_Cl_2_ 298 K	583	—	2.1	—	—	0.13

L1	303 (8.7), 309 (9.7), 316 (11.9), 341 (4.1)	CH_2_Cl_2_ 298 K	412	—	1.0	—	—	0.07

L2	260 (15.9), 326 (4.7), 405 (24.0, br), 417 (24.7)	CH_2_Cl_2_ 298 K	455	—	3.6	—	—	0.94

L3	333 (14.6), 350 (22.4), 367 (20.6)	CH_2_Cl_2_ 298 K	378, 398, 419 (sh)	—	0.5	—	—	0.11

L4	321 (3.9, br), 376 (5.3, br), 486 (14.8, sh), 517 (45.4)	CH_2_Cl_2_ 298 K	533	—	6.4	—	—	0.83

^*a*^Data were obtained from steady-state measurements with degassed CH_2_Cl_2_ solutions (2 × 10^–5^ M) unless specified. Measurements with glassy solutions were performed in EtOH/MeOH (4 : 1) mixture at 77 K.

^*b*^Emission lifetimes of prompt fluorescence (*τ*_PF_) were determined by time-correlated single photon counting (TCSPC) measurement.

^*c*^Emission lifetimes of delayed fluorescence (*τ*_DF_) were obtained from fitting the decay of the time-resolved emission (TRE) as a mono-exponential decay in the delay time range of 0–40 ns and 1–46 μs, respectively. Measurements were performed in degassed CH_2_Cl_2_ (5 × 10^–5^ M) solutions.

^*d*^Emission quantum yields (*Φ*_em_) were obtained using quinine sulfate in degassed 0.5 M H_2_SO_4_ (*Φ* = 0.546) as the standard unless specified. *Φ*_em_ measured in steady state is the overall emission quantum yield, *i.e. Φ*_em_ = *Φ*_PF_ + *Φ*_DF_ for **1a–3a** and **5a–6a**.

^*e*^Obtained from time-resolved emission spectra.

^*f*^Emission lifetime was not determined (n.d.) due to weak emission signal.

^*g*^Determined from time-resolved emission spectra in degassed CH_2_Cl_2_ (1 × 10^–5^ M) solutions.

^*h*^Determined from fs-TRF spectra.

^*i*^Emission quantum yields (*Φ*_em_) were obtained using [Ru(bpy)_3_][PF_6_]_2_ in degassed acetonitrile as the standard (*Φ* = 0.062).

#### Electronic absorption of Au(i) complexes **1a–6a**

**Fig. 3:**
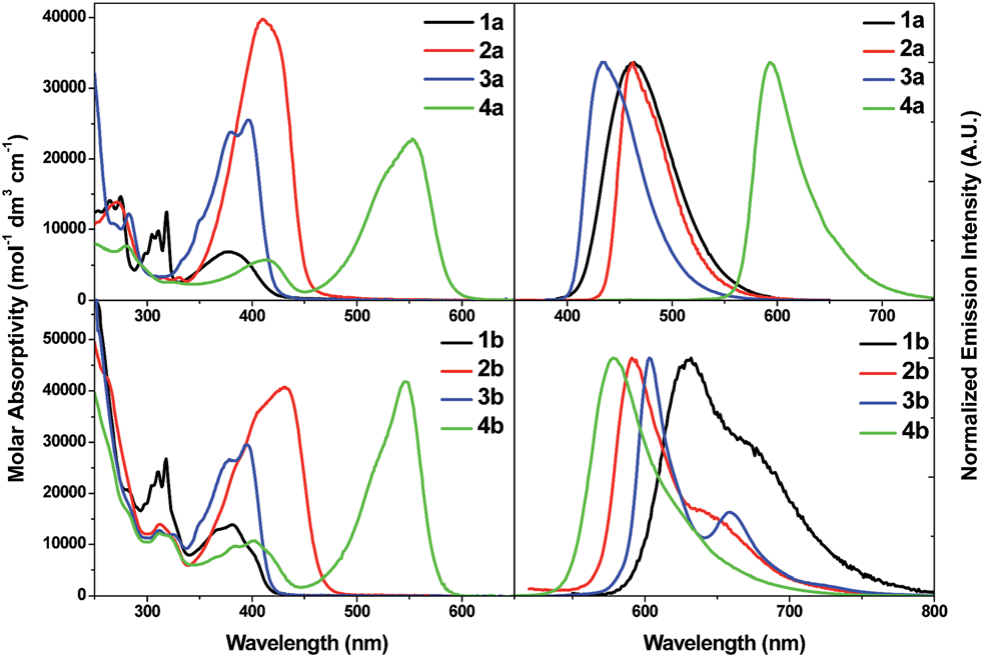
UV-vis absorption spectra (left) and emission spectra (right) of **1a–4a** (top) and **1b–4b** (bottom) in CH_2_Cl_2_ at 298 K (2 × 10^–5^ M).

Fig. 3 (top left) shows the absorption spectra of **1a–4a**. The lowest energy absorption bands of **1a–4a** are at 379, 410, 397 and 553 nm, respectively, and their molar absorptivities (*ε*) fall in the range 6.9 × 10^3^ to 4 × 10^4^ mol^–1^ dm^3^ cm^–1^. These lowest energy absorption bands have spectral features resembling those of L1–L4 (Fig. S4 in ESI†) and are attributable to the dipole-allowed intraligand transitions of the arylacetylide ligands (^1^ππ*(C≡CR)) with some charge-transfer character. Similar assignments were also made for other Au(i) alkynyl complexes in the literature.^6,7,8a^ Bathochromic shifts of ^1^IL transitions of arylacetylides are observed upon coordination of the arylacetylides to the Au(i) ion and are ascribed to π-interaction between Au(i) 5d orbitals and the ligand π-orbitals (see MO surfaces in Fig. 9 and 10).

Replacing the neutral auxiliary ligand PCy_3_ in **1a** with 2,6-dimethylphenyl isocyanide (RNC, **5a**) and 1,3-dimethylimidazol-2-ylidene (NHC, **6a**) results in a slight change in *λ*_max_ of the lowest energy absorption band (*λ*_max_ = 371 nm (**5a**, RNC) and 383 nm (**6a**, NHC) *cf*. *λ*_max_ = 379 nm (**1a**, PCy_3_)) (Fig. S6 in ESI†).

#### Electronic absorption spectra of Au(iii) complexes **1b–4b**

Fig. 3 (bottom left) shows the UV-vis absorption spectra of the Au(iii) complexes studied in the present work. The spectral features at *λ* > 300 nm are similar to those of the Au(i) analogues and free ligands L1–L4. Complexes **1b–4b** display low-energy absorption bands at *λ*_max_ = 381, 432, 395 and 546 nm, respectively, and with *ε* values in the range of 1.38 × 10^4^ to 4.17 × 10^4^ mol^–1^ dm^3^ cm^–1^. These absorption bands are, like the Au(i) complexes, attributable to ^1^ππ*(C≡CR) transitions. Comparisons of the absorption spectra of **1a–4a** and **1b–4b** at *λ* ≤ 300 nm revealed that the absorption bands in this spectral region are more intense in **1b–4b** with *ε* values of *ca.* 4 × 10^4^ mol^–1^ dm^3^ cm^–1^ and these high energy absorption bands likely involve intraligand ^1^ππ*(C^N^C) transitions (Fig. S7 in ESI†).

### Steady-state emission spectroscopy

All of the complexes are luminescent in degassed CH_2_Cl_2_ at room temperature and in 77 K glassy solutions (EtOH : MeOH = 4 : 1) upon excitation at the corresponding lowest-energy absorption *λ*_max_. As depicted in Table 2, there is a distinct difference between the two classes of complexes: the Au(i) complexes **1a–6a** display predominantly fluorescence while the Au(iii) complexes, **1b–3b**, exhibit exclusively weak phosphorescence. Complex **4b**, on the other hand, shows fluorescence only.

#### Emission of **1a–4a** and **5a–6a**

In dichloromethane solutions, structureless emission bands are observed at *λ*_max_ = 467, 466, 439 and 553 nm for **1a–4a**, respectively (Fig. 3, top right). The corresponding excitation spectra of **1a–4a** can be found in the ESI (Fig. S8†). The emission quantum yields for **1a–3a** are high (*Φ*_em_ = 0.91, 0.70 and 0.78, respectively). In the case of **4a**, its emission quantum yield is low (*Φ*_em_ = 0.04). Emission lifetimes of **1a–4a** are in the nanosecond time regime: 0.8–12 ns. As the emissions of these Au(i) complexes resemble those of the corresponding free ligands L1–L4, they are attributable to ^1^ππ*(C≡CR) excited states, with some charge transfer character, which probably arise from mixings of metal-to-ligand charge-transfer (MLCT) character. Solvent effects on the emissions of **1a–3a** can be found in Fig. S10, ESI.† There is no discernible phosphorescence for **1a–4a** under steady-state conditions in solutions at either room temperature or 77 K (Fig. S9, ESI†).

Comparing the three Au(i) complexes bearing the benzothiadiazole moiety, the emission energies (*λ*_max_ = 467, 456 and 476 nm for **1a** (PCy_3_), **5a** (RNC) and **6a** (NHC), respectively (Fig. S11 in ESI†)) and emission lifetimes (*τ*_PF_ ∼ 11–14 ns) are similar, indicating that the auxiliary ligand plays an insignificant role in modification of the electronic structures of the excited states.

#### Emission of **1b–4b**

Emission spectra of the Au(iii) complexes are depicted in Fig. 3 (bottom right). Contrary to the Au(i) analogues where the emission profiles are structureless, the emission spectra of complexes **1b–3b** are vibronically structured with *λ*_max_ at 630, 592 and 603 nm and quantum yields of 0.003, 0.01 and 0.04, respectively. The emission lifetimes are of hundreds of microseconds (∼100 μs for **1b** and **2b**; ∼200 μs for **3b**). Taking into account the large Stokes shifts (between 6300 and 10 400 cm^–1^), structured emission profiles, and long emission lifetimes, the emissions of **1b–3b** could be attributed to ^3^ππ*(C≡CR) excited states with negligible mixings of MLCT and LLCT character (LLCT = ligand-to-ligand charge transfer). Solvent effects on the emissions of **1b** can be found in Fig. S12, ESI.† On the contrary, **4b** shows emission with a small Stokes shift of 920 cm^–1^ and emission lifetime of only 2.1 ns. Thus, the emission of **4b** is derived from fluorescence with ^1^ππ*(C≡CBodipy) parentage.

### Time-resolved spectroscopies for the gold(i) complexes

**Fig. 4:**
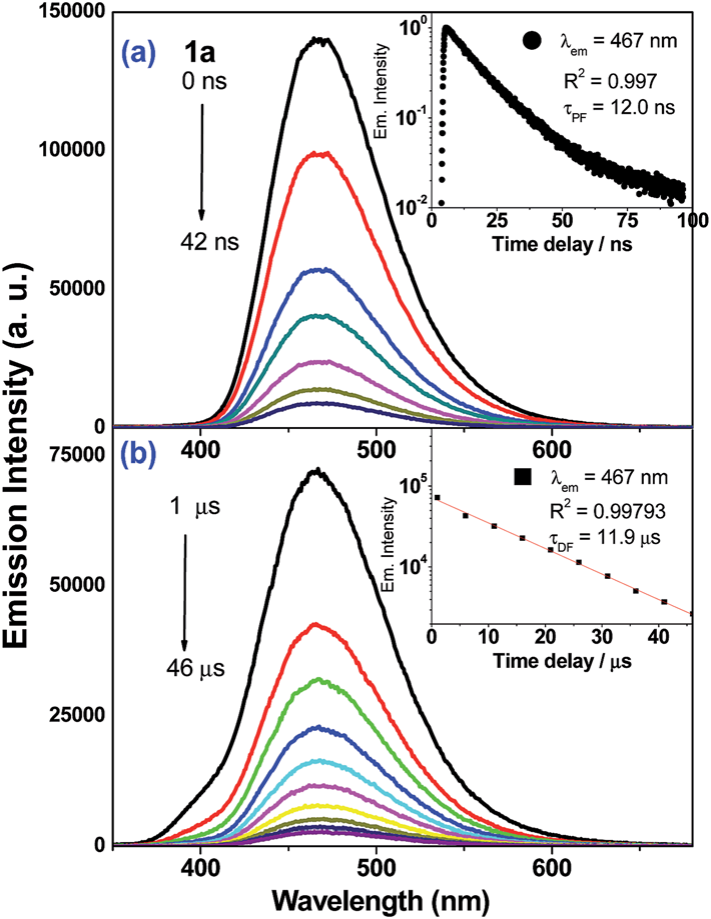
ns-TRE spectra of **1a** recorded from (a) 0–42 ns and (b) after a time delay of 1 μs in degassed CH_2_Cl_2_ (5 × 10^–5^ M) at 298 K. Inset shows the emission kinetic decay trace. Decay time constants were fitted as mono-exponential decay (*λ*_exc_ = 355 nm).

Nanosecond time-resolved emission (ns-TRE) spectra of the Au(i) complexes in degassed CH_2_Cl_2_ solutions at 298 K (5 × 10^–5^ M) are measured at different time delays and are presented in Fig. 4 (**1a**) and ESI (**2a–3a**, **5a–6a**; Fig. S13†). There are two components in the emission decay: a major component which decays within nanoseconds (*τ*_1_ = 12.0 (**1a**), 2.7 (**2a**), 2.8 (**3a**), 11.0 (**5a**) and 14.0 ns (**6a**)) and a minor component with microsecond decay lifetime (*τ*_2_ = 11.9 (**1a**), 4.6 (**2a**), 2.6, 12.5 (**3a**), 6.8 (**5a**) and 7.4 μs (**6a**)). For each of these Au(i) complexes, both decay components have identical emission profile and peak energy and so, the short-lived one (*τ*_1_) is assigned to be prompt fluorescence (PF) while the long-lived one (*τ*_2_) is delayed fluorescence (DF) of ^1^ππ*(C≡CR) character. In the case of **4a**, only PF (*τ*_PF_ = 0.8 ns) is observed. The proportion of DF and PF constituting the total emission of **1a–3a** have been estimated (Table 3): the intensity of DF is minute (<3%) when compared with that of PF (>97%). It is noted that delayed fluorescence in the microsecond time regime is indicative of the emission generated from a long-lived excited state. This is further supported by nanosecond transient absorption (ns-TA) measurements that reveal the presence of long-lived absorbing species in the microsecond timescale (*vide infra*).

**Table 3:** Proportion of PF and DF constituting the fluorescence of **1a–3a** ^*a*^

Complex	% PF	% DF
**1a**	99.2	0.81
**2a**	99.9	0.1
**3a**	97.1	2.9

^*a*^% PF and % DF are estimated by integrating the emission intensity of degassed CH_2_Cl_2_ (5 × 10^–5^ M) in the spectral region of *λ* = 350–700 nm over the time range: 0–500 ns and 800 ns to 999 μs, respectively (*λ*_exc_ = 355 nm).

**Fig. 5:**
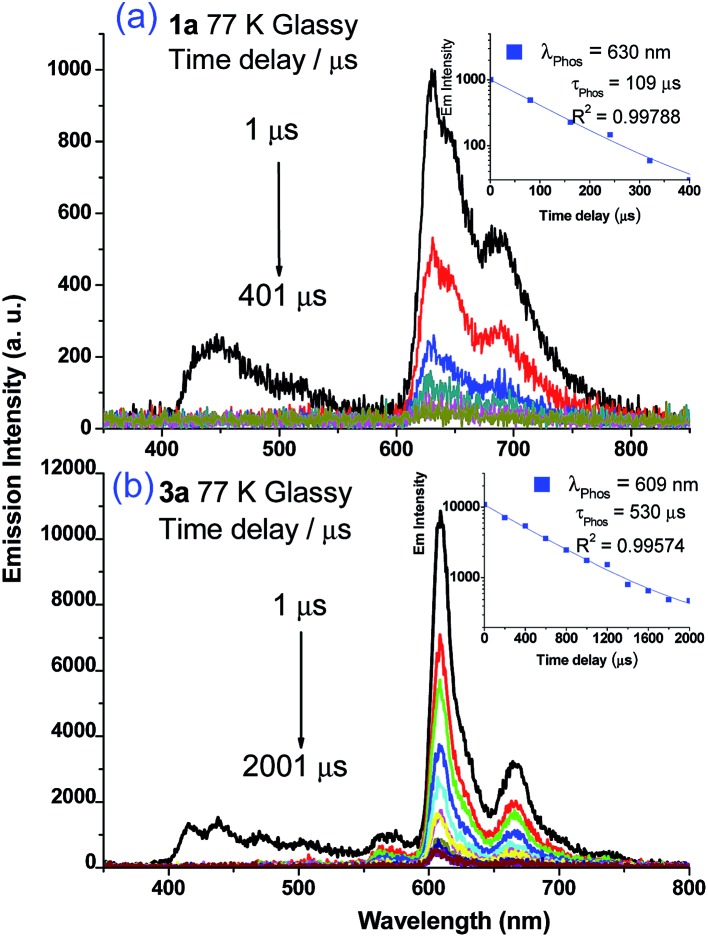
ns-TRE spectra of (a) **1a** and (b) **3a** in 77 K glassy solution (EtOH/MeOH = 4 : 1) recorded at different time intervals. *λ*_exc_ = 355 nm; integration time: 80 and 200 μs for **1a** and **3a**, respectively. Insets of (a) and (b) show the kinetic decay traces at the specified wavelengths with the estimated phosphorescence lifetime (*τ*_phos_).

**Fig. 6:**
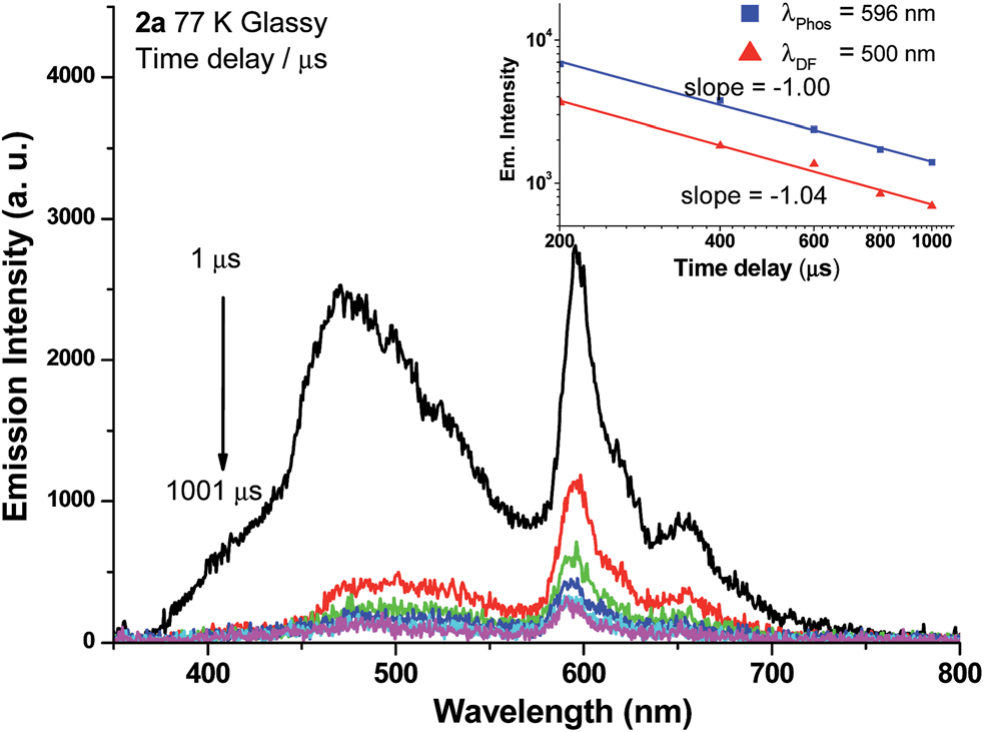
ns-TRE spectra of **2a** in 77 K glassy solution (EtOH/MeOH = 4 : 1) recorded at different time intervals. *λ*_exc_ = 355 nm; integration time: 200 μs. Inset shows the log–log plot of emission intensity of DF (*λ*_DF_ = 500 nm) and phosphorescence (*λ*_phos_ = 596 nm) of **2a** in 77 K glassy solution against time; both decay according to a power law: *I* ∝ *t* ^–1^.

Weak phosphorescence bands were observed for **1a–3a** under different conditions. For dilute CH_2_Cl_2_ solutions (1 × 10^–5^ M) at 298 K, dominant emissions were observed in the spectral region of 440–470 nm, which correspond to fluorescence (Fig. S14, left panel in ESI†). In addition, weak emission peaks at *ca.* 600 nm become discernible for **2a** and **3a** and the lifetimes measured are 13.6 and 61.9 μs, respectively (Fig. S13, right panel in ESI†). Cooling to 77 K gives more resolved phosphorescence bands with vibrational progression spacings of 1300–1400 cm^–1^ for all three complexes (Fig. 5 and 6). For **1a**, contrary to the ns-TRE spectra recorded in degassed CH_2_Cl_2_ at room temperature (Fig. 4 (5 × 10^–5^ M); Fig. S14, ESI† (1 × 10^–5^ M)) where only DF could be observed over the time range 1–46 μs, in 77 K glassy solution, phosphorescence at 630 nm is dominant and the weak DF at 467 nm vanishes after 80 μs (Fig. 5a). The phosphorescence band decays with first-order kinetics at *τ*_phos_ = 109 μs. Similarly, the low-temperature ns-TRE spectra of **3a** is dominated by phosphorescence at 609 nm and DF vanishes after 200 μs (Fig. 5b). The phosphorescence band also decays mono-exponentially with *τ*_phos_ = 530 μs. The photodynamics of **2a** at 77 K, however, is different from that of **1a** and **3a**: both DF and phosphorescence of **2a** are of comparable intensities initially (∼1 μs) in the 77 K ns-TRE spectra (Fig. 6); in addition, DF and phosphorescence do not follow first-order kinetics but decay according to the power law (*I* ∝ *t* ^–1^) in the time interval 1 μs to 1.2 ms (inset of Fig. 6). The thermally induced Stokes shifts (Δ*E*_s_ = *E*_00_ (77 K) – *E*_00_ (298 K)), being ∼0 (**2a**) and ∼107 cm^–1^ (**3a**), are small, thus supporting that the phosphorescence bands are originated from ^3^IL.^15^ Moreover, as the emission energies and profiles of the low-energy bands are similar to those of the steady-state emission spectra of the Au(iii) analogues, the low-energy emission bands of **1a–3a** are assigned to be from phosphorescence decay of the ^3^ππ*(C≡CR) excited state.

**Fig. 7:**
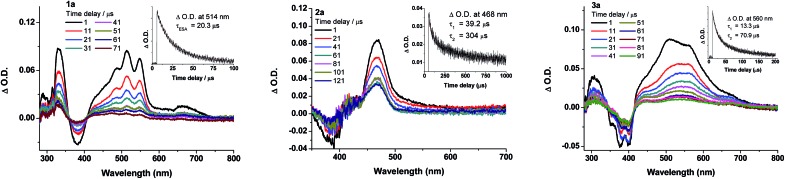
Nanosecond transient absorption (ns-TA) difference spectra of **1a–3a** recorded at selected decay times in degassed CH_2_Cl_2_ (5 × 10^–5^ M) at 298 K. Insets show the ESA kinetic decay trace at the specified wavelengths; decay lifetimes were fitted as mono-exponential decay for **1a** and bi-exponential decays for **2a** and **3a**. (*λ*_exc_ = 355 nm; integration time: 200 ns).

Nanosecond transient absorption (ns-TA) difference spectra of **1a–3a** (Fig. 7) and **5a–6a** (Fig. S15 in ESI†) have been recorded in deoxygenated CH_2_Cl_2_ at a gate delay of 1 μs after excitation at *λ* = 355 nm. The ns-TA spectra are characterized by an intense positive signal due to excited-state absorption (ESA) within the spectral range 400–700 nm. The decay time constants of the lowest-energy ESA (*τ*_ESA_) are 20.3 (**1a**), 39.2; 304 (**2a**), and 13.3; 70.9 μs (**3a**) (insets of Fig. 7). Changing the auxiliary ligand from PCy_3_ (**1a**) to RNC (**5a**) and NHC (**6a**) results in negligible changes in the ns-TA spectra and *τ*_ESA_ (Fig. 7 *vs.* S15†), suggesting that auxiliary ligand has little effect on the photophysics of the gold(i) arylacetylide complexes.

### Time-resolved spectroscopies for the gold(iii) complexes

**Fig. 8:**
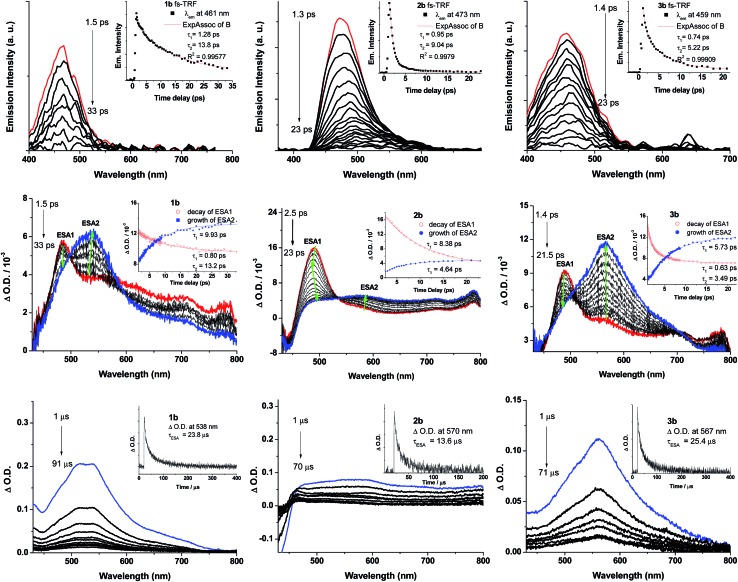
(Top) fs-TRF spectra and (middle) fs-TA difference spectra of **1b–3b** in CH_2_Cl_2_ (5 × 10^–5^ M) at 298 K (*λ*_exc_ = 400 nm; 120 fs fwhm). Arrows indicate the spectral evolution. (Bottom) ns-TA difference spectra of **1b–3b** in degassed CH_2_Cl_2_ (laser *λ*_exc_: 355 nm). Insets show the kinetic time profiles and the decay time constants at the specified wavelengths.

ns-TRE and ns-TA difference spectra of **1b–3b** have been recorded in degassed CH_2_Cl_2_ solutions at 298 K at a gate delay of 1 μs. The ns-TRE spectra of **1b–3b** (Fig. S16 in ESI†) have the same emission profiles and peak positions as the corresponding steady-state phosphorescence spectra and exhibit single exponential decay lifetimes of 20.8 (**1b**), 9.7 (**2b**) and 18.3 μs (**3b**). For the ns-TA difference spectra of **1b–3b** (Fig. 8, bottom panel), a broad positive ESA band was observed in the spectral region 450–800 nm; this ESA signal follows first-order kinetics with lifetimes determined to be 23.8 (**1b**), 13.6 (**2b**) and 25.4 μs (**3b**), in reasonable agreement with the phosphorescence decay lifetimes determined from their respective ns-TRE spectra, thus indicating that the broad ESA is derived from T_1_ → T_*n*_ absorption.

To probe the early excited state dynamics of the gold(iii) complexes, in particular the events associated with ISC, femtosecond time-resolved fluorescence (fs-TRF) and transient absorption difference spectra (fs-TA) of **1b–3b** have been recorded. Fig. 8 depicts the fs-TRF (top panel) and fs-TA spectra (middle panel) of complexes **1b–3b** in CH_2_Cl_2_ solution at various time intervals after 400 nm excitation at 298 K. Promptly (<2 ps) after photo-excitation, an unstructured fluorescence band peaking at 461 (**1b**), 473 (**2b**) and 459 nm (**3b**) appears and decays completely within 100 ps. As the TRF emission peaks and profiles closely resemble those of their Au(i) analogues, **1a–3a**, these TRF spectra are suggested to be originated from the ^1^ππ*(C≡CR) excited state. Fitting of the kinetic traces at their peaking wavelengths reveals that bi-exponential functions are required for **1b–3b** with *τ*_1_ and *τ*_2_ being 1.28 and 13.8 ps for **1b**, 0.95 and 9.04 ps for **2b**, and 0.74 and 5.22 ps for **3b**.

In the fs-TA of **1b–3b** (Fig. 8, middle panel), all three complexes displayed similar spectral transformations: the initially formed (∼1.4–2.5 ps) excited state absorption peaking at ∼490 nm (ESA1) decays with a concomitant growth of a broad band covering a spectral region 450–800 nm (ESA2) and is fully developed within 40 ps and persists up to 2.7 ns (the longest time recorded in the fs measurements). Clear isosbestic points could be observed at ∼500 nm (**1b**), 530 nm (**2b**) and ∼500 and 700 nm (**3b**) during the temporal evolution. Such kind of spectral conversion points to a precursor–successor relationship between ESA1 and ESA2. Kinetic analyses at representative wavelengths of these TA spectra reveals that ESA1 of **1b** and **3b** decay bi-exponentially with *τ*_1_ and *τ*_2_ being 0.80 and 13.2 ps for **1b** and 0.63 and 3.49 ps for **3b**, respectively, whereas ESA1 of **2b** decays with a single exponential time constant of *τ*_2_ = 8.38 ps. ESA2, on the other hand, grows with first-order kinetics for all three complexes **1b–3b** with time constants *τ*_ESA2_ = 9.93 (**1b**), 4.64 (**2b**) and 5.73 ps (**3b**). Given the similar decay time constants between the fs-TRF and ESA1 in fs-TA of **1b–3b**, the spectral dynamics for both time-resolved spectra should be originated from the same S_1_ excited state, namely, the ^1^ππ*(C≡CR) excited state as revealed in the fs-TRF. On the other hand, comparing the ESA2 in fs-TA spectra at the longest time recorded with the corresponding ns-TA spectra for each Au(iii) complex (Fig. 8, bottom panel), the two spectra are similar, indicating that ESA2 is derived from T_1_ → T_*n*_ absorption. Because there is a precursor–successor relationship between the ESA1 (S_1_ → S_*n*_ absorption) and ESA2 (T_1_ → T_*n*_ absorption), *τ*_2_ of ESA1 is assigned to ISC from the S_1_ excited state to a receiving triplet excited state, which then internally converted to the T_1_ excited state with an ultrafast time scale. Thus, *τ*_ISC_ = 13.2 ps (**1b**), 8.38 ps (**2b**) and 3.49 ps (**3b**). The short *τ*_1_ = 0.80/1.28 (**1b**), 0.95 ps (**2b**) and 0.63/0.74 ps (**3b**) of ESA1/TRF may likely correspond to the S_1_ vibrational relaxation.

### Intersystem crossing rate

The spectroscopically determined intersystem crossing rate constants (*k*_ISC_) and the corresponding time constants (*τ*_ISC_) for both Au(i) and Au(iii) complexes studied herein are tabulated in Table 4. For **1a–3a** and **5a–6a**, assuming that the major non-radiative decay of the S_1_ excited state is ISC, *i.e. k*_nr_ ≈ *k*_ISC_, an upper bound approximation of the *k*_ISC_ values could be obtained by eqn (1):
1

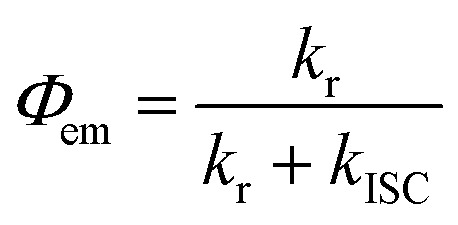




**Table 4:** *k*_ISC_ and *τ*_ISC_ of the Au(i) and Au(iii) complexes

Complex	*k*_ISC_ ^*a*^/10^7^ s^–1^	*τ*_ISC_ ^*b*^/ns	Complex	*k*_ISC_ ^*b*^/10^10^ s^–1^	*τ*_ISC_ ^*c*^/ps
**1a**	<0.75	133	**1b**	7.57	13.2
**2a**	<11.1	9.0	**2b**	11.9	8.38
**3a**	<7.9	12.7	**3b**	28.7	3.49
**5a**	<0.91	110			
**6a**	<1.14	87.5			

^*a*^ *k*_ISC_ for **1a–3a**, **5a–6a** are calculated according to eqn (1).

^*b*^ *τ*_ISC_ = 1/*k*_ISC_.

^*c*^ *τ*_ISC_ of **1b–3b** is estimated from *τ*_2_ obtained from the fs-TA spectra of ESA1.

The estimated *k*_ISC_ for the gold(i) complexes are 7.5 × 10^6^ to 1.1 × 10^8^ s^–1^ and the intersystem crossing time constants (*τ*_ISC_) are 9.0–133 ns. These *τ*_ISC_ are much larger than those of many phosphorescent transition-metal complexes (*τ*_ISC_ in the femtosecond to picosecond timescale). For **1b–3b**, the *τ*_ISC_ values are more than three orders of magnitude faster than their gold(i) analogues; these ISC rates, nevertheless, are comparable to other transition-metal complexes where S_1_ → T_1_ ISC is mediated by a higher-lying T_*n*_ triplet excited state.^16,17^

## Computational study

The different luminescence behaviors between the Au(i) and Au(iii) systems were investigated by DFT/TDDFT calculations. The pair (**1a**, **1b**) was chosen as a representative example to examine why the Au(i) complexes studied herein display only fluorescence while the Au(iii) counterparts exhibit exclusively phosphorescence. As the Bodipy-functionalized complexes give fluorescence for both Au(i) and Au(iii) complexes, DFT/TDDFT calculations were also performed on the pair, (**4a**, **4b**). To save computational time, the cyclohexyl groups of the phosphine ligands in **1a** and **4a** were replaced by methyl groups.

### Calculations on **1a** and **1b**

**Fig. 9:**
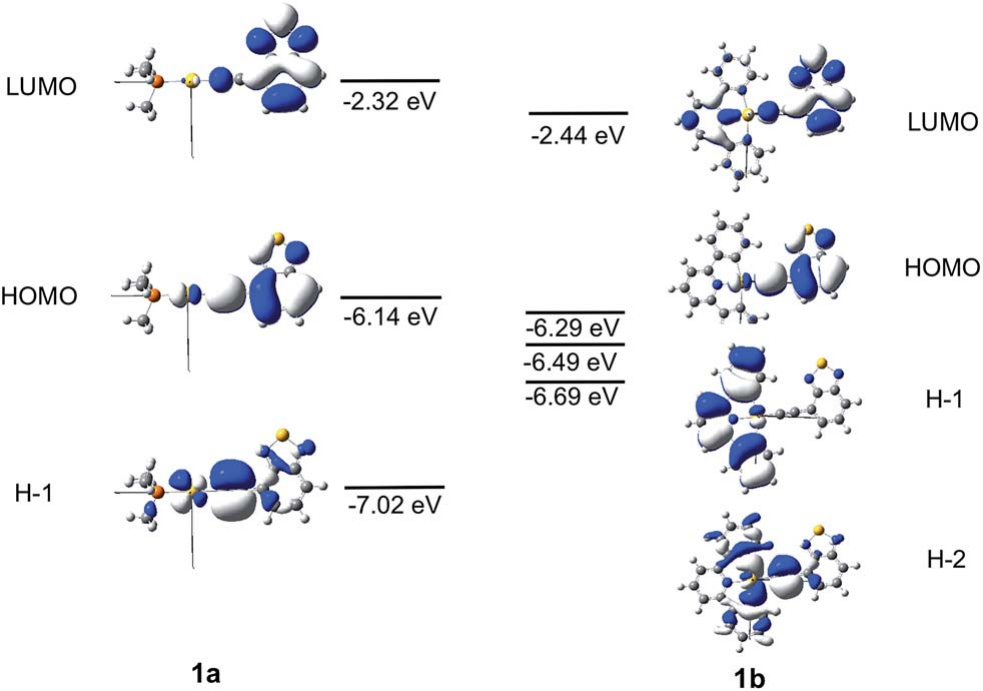
Frontier MOs of **1a** and **1b** at the optimized S_0_ geometries. Orbital energies are also given in eV.

The frontier MO diagrams of **1a** and **1b** are shown in Fig. 9. The HOMO and LUMO for both complexes **1a** and **1b** are predominantly localized on the arylacetylide ligand. For **1b**, a considerable contribution (17%) from the C^N^C moiety to the LUMO is also noted. The energy gap between HOMO and H–1 in **1a** is approximately 0.88 eV. For **1a**, the H–1 is comprised of the antibonding combinations of Au(d_*xy*_) and π(C≡C) orbitals with little involvement of the phosphine ligand. For **1b**, H–1 is composed of Au(d_*xz*_) and the π(C^N^C) orbitals; the energy gap between HOMO and H–1 in **1b** is only 0.2 eV. The H–2 of **1b** is made up of an antibonding combination of the Au(d_*xy*_), π(C≡C) and σ(C^N^C) orbitals, with a HOMO/H–2 orbital energy gap of only ∼0.4 eV. Clearly, the cyclometalated [C^N^C] ligand has a role in destabilizing the Au(d) orbitals. Therefore, the HOMO and H–1/H–2 energy gaps in **1b** are much smaller than that in **1a**.

The energies of the singlet and triplet excited states and the associated nature and composition for **1a** and **1b** at their respective optimized singlet ground state geometries are obtained by TDDFT and are shown in Table S5 and S6 in ESI.† For **1a**, there is only one triplet excited state (T_1_) which is more than 10 000 cm^–1^ below S_1_. In addition, both S_1_ and T_1_ excited states are of the same parentage and are derived from HOMO → LUMO transition (∼90%) and thus, there would be no effective SOC between them. The triplet excited states above S_1_ were also considered; the closest lying T_*m*_ excited state with efficient SOC is when *m* = 4, which is derived from a H–1 to LUMO transition (90% H–1 → L). However, the energy separation Δ*E*(S_1_–T_4_) is –3180 cm^–1^, which is too large to be overcome by thermal activation.

On the other hand, for **1b**, there are four triplet excited states which are lower-lying than S_1_, of which the closest-lying T_4_ excited state is only ∼70 cm^–1^ below the S_1_ excited state. Thus, thermal energy at room temperature assists facile ISC, even though SOC is small between the S_1_ and T_4_ excited states (|<S_1_|*H*_SOC_|T_4_>|^2^ ∼ 1.5 cm^–2^). In addition, among the triplet excited states above S_1_, there is a close-lying T_5_ excited state derived from the H–2 → LUMO transition (79%) which lies only 390 cm^–1^ above the S_1_ excited state and ISC from S_1_ to T_5_ could be thermally activated. Besides, owing to the different orientations of the d-orbitals in HOMO and H–2, the S_1_ and T_5_ excited states could have effective SOC (|<S_1_|*H*_SOC_|T_5_>|^2^ ∼ 4.1 × 10^3^ cm^–2^).

### Calculations on **4a** and **4b**

**Fig. 10:**
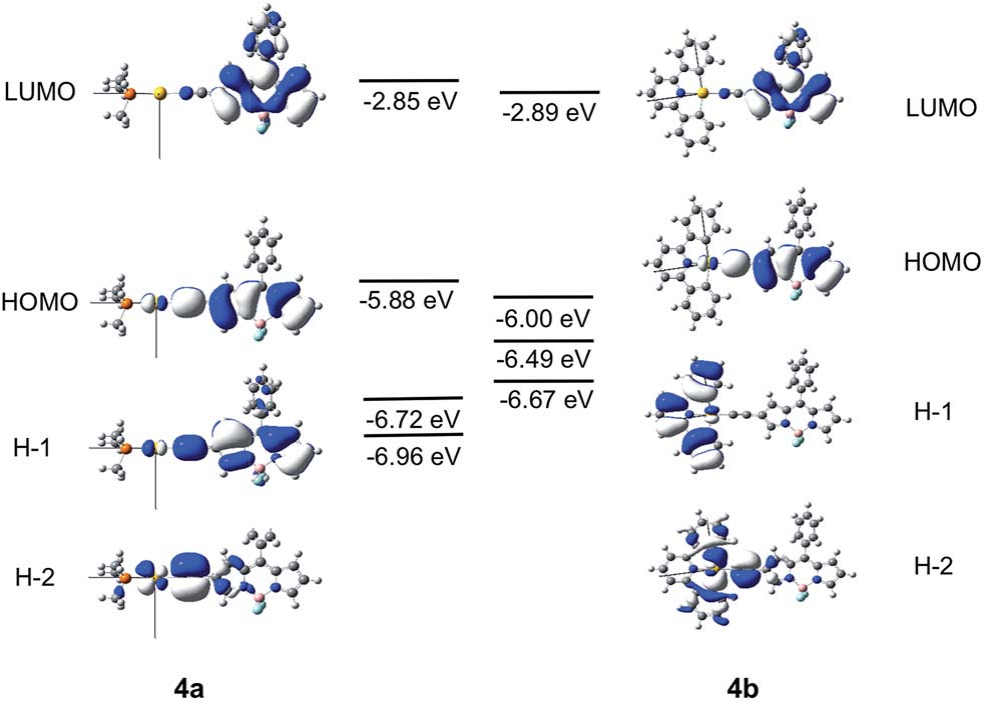
Frontier MOs of **4a** and **4b** at their optimized S_0_ geometries. Orbital energies are also given in eV.

The Frontier MOs for **4a** and **4b** are shown in Fig. 10. Relative to **1a** and **1b**, the HOMO is destabilized and the LUMO is stabilized for **4a** and **4b**. Even for **4b**, which contains a cyclometalated [C^N^C] ligand, the LUMO is predominantly localized on the Bodipy-functionalised arylacetylide ligand. The HOMO is composed of an antibonding combination of the Au(d_*yz*_) and π(C≡CBodipy) orbitals. The energies and compositions of the singlet and triplet excited states of **4a** and **4b** at their respective optimized singlet ground state geometries were obtained by TDDFT and are collected in Tables S7 and S8 in ESI.† For this pair, (**4a**, **4b**), the two triplet excited states, T_1_ and T_2_, are more than 2000 cm^–1^ below the S_1_ excited state and are all composed of Au(d_*yz*_) orbitals. As SOC between the coupling singlet and triplet excited states would be ineffective with d-orbitals of the same orientation, ISC from S_1_ to T_2_ (or T_1_) for **4a** and **4b** would be sluggish.

For **4a**, the T_3_ excited state is the closest-lying triplet excited state that could have effective SOC with the S_1_ excited state due to a minor contribution of the H–5 → LUMO transition to the T_3_ excited state (H–5 is composed of the Au(d_*z*_^2^) orbital); however, Δ*E*(S_1_–T_3_) is –2955 cm^–1^ (negative sign indicates that T_3_ lies above S_1_) which is much larger than the thermal energy. For **4b**, the T_3_ excited state is also the closest-lying triplet excited state that could have effective SOC with the S_1_ excited state due to a minor contribution of H–1 → LUMO transition in the T_3_ excited state (the d-orbitals of the Au(iii) ion at the HOMO and H–1 of **4b** are of different orientations, Fig. 10). However, the singlet–triplet gap, Δ*E*(S_1_–T_3_) = –1192 cm^–1^, is also much larger than the thermal energy. Thus, the pair (**4a**, **4b**) is expected to have slow ISC rates, when taking into consideration both the singlet–triplet energy gaps and SOC.

## Discussion

### General remarks on the photophysical properties

The emissions of the Au(i) complexes, **1a–6a** are attributable to ^1^IL ππ*(C≡CR) excited states. Pronounced red shifts in emission *λ*_max_ of arylacetylides can be observed upon their coordination to Au(i) ion (*e.g. λ*_max_ = 412 nm (L1) *vs.* 467 nm (**1a**)). Considering the complexes **1a**, **5a** and **6a**, which have different neutral auxiliary ligands (phosphine (PCy_3_, **1a**), isocyanide (RNC, **5a**) and N-heterocyclic carbene (NHC, **6a**)) but the same acetylide ligand with a benzothiadiazole moiety, the lowest energy emission *λ*_em_ red shifts with the auxiliary ligand from 456 nm (RNC) to 467 nm (PCy_3_) to 476 nm (NHC). A rationalization would be that the NHC, being the strongest σ-donor ligand among the auxiliary ligands in the three complexes, destabilizes the Au(d) orbital to the greatest extent. From DFT calculations, the HOMO is comprised of a Au(d) orbital and π(C≡CR) (Fig. 9). Thus, the more electron-donating the auxiliary ligand, the more destabilized the HOMO, and hence, the smaller the HOMO–LUMO gap and the ^1^ππ*(C≡CR) energy. A similar trend in the lowest energy absorption *λ*_abs_ can also be observed on changing the auxiliary ligand from RNC (371 nm) to PCy_3_ (379 nm) and NHC (383 nm).

Most of the reported luminescent cyclometalated Au(iii) complexes display phosphorescence that comes from the ^3^ππ* IL excited state localized on the cyclometalated ligands.^13^ For the Au(iii) complexes studied herein, **1b–3b**, the lowest-energy triplet excited states are of ^3^ππ*(C≡CR) in nature, with *λ*_max_ = 630, 592 and 603 nm respectively. These complexes, however, exhibit rather weak phosphorescence, with *Φ*_em_ values in the range of 0.003–0.04. It is noted that in 77 K glassy solutions, the phosphorescence lifetimes are significantly increased compared with those obtained in degassed CH_2_Cl_2_ at RT (*e.g.* 205 μs at RT to 2.2 ms at 77 K for **3b**). Since low temperature and rigid glassy matrix can impede structural distortion, the lifetimes obtained at 77 K could reflect the intrinsic radiative lifetime of the complexes. The especially long emission lifetimes can reflect the predominant localization of the emitting T_1_ excited state on the arylacetylide ligand, *i.e.* ^3^ππ*(C≡CR) with little participation of the metal ion. This is also corroborated by the small thermally induced Stokes shifts (Δ*E*_s_ = *E*_00_ (77 K) – *E*_00_ (298 K)) of less than 600 cm^–1^ (Table 2).

### Intersystem crossing in gold(i) and gold(iii) complexes

ISC is usually fast in transition-metal complexes with time constants (*τ*_ISC_) in the fs to ps time regimes. In the literature, there are numerous examples which show ultrafast ISC,^1,18–25^ *e.g.* [M(bpy)_3_]^2+^ (M = Ru or Fe, *τ*_ISC_ = 30 fs),^19a,b^ [Re(L)(CO)_3_(bpy)] (*τ*_ISC_ = 100–140 fs),^20^ [Ir(piq)_3_] (piq: 1-phenylisoquinoline; *τ*_ISC_ = 70 fs),^21*b*^ [Pt(PBu_3_)_2_(C≡CPh)_2_] (*τ*_ISC_ = 70 fs),^22^ and [Cy_3_PAu(2-naphthyl)] (*τ*_ISC_ = 230 fs)^23^ *etc.* These *τ*_ISC_ correspond to rates of intersystem crossing (*k*_ISC_) in the range of 10^12^ to 10^13^ s^–1^. The fast *k*_ISC_ in transition-metal complexes is traditionally attributed to a large spin–orbit coupling (SOC) constant inherited from the heavy metal atom. However, there are increasing number of reports revealing slow ISC rate (*k*_ISC_ ∼ 10^8^ s^–1^) in spite of the presence of heavy transition metal, such as the cases of Rh(i)- and Ir(iii)-bis(arylethynyl)cyclopentadiene,^3^ Au(i)-pyrene,^4*b*^ Pt(ii)-perylene/^4*c*^ tetracene,^4*e*^ and Pd(ii)-perylene diimide;^4*f*^ all these complexes contain highly conjugated ligand systems and display ligand-dominated ^1^ππ* fluorescence. There are also cases where comparable *k*_ISC_ and *k*_r_ of S_1_ → S_0_ leads to the observation of dual fluorescence–phosphorescence under steady-state condition, *e.g.*, [Pt(L)(acac)] and [Ir(L)(acac)] (L = 2-(oligothienyl)pyridine);^26^ [Os(L)(CO)_3_X] (L = 8-quinolinolate^27^ or isoquinoline-triazole),^28^ and [Bu_4_N]_4_[Pt_2_(μ-P_2_O_5_(BF_2_)_2_)_4_],^29^ *etc.*

In this work, the luminescence behaviour of the Au(i) and Au(iii) complexes are drastically different, even though they have the same metal and arylacetylide ligands. Ligand-dominated fluorescence has been observed with the Au(i) complexes, **1a–3a** and **5a–6a**, with *k*_ISC_ estimated to range from 7.5 × 10^6^ to 1.1 × 10^8^ s^–1^. The Au(iii) complexes **1b–3b**, on the other hand, display phosphorescence, with *k*_ISC_ estimated to be larger than 10^10^ s^–1^. The major difference between the two series of gold complexes is the oxidation state of Au ion, that dictates the coordination geometry, *i.e.* a linear geometry for the Au(i) complexes, **1a–6a**, and a square-planar geometry for Au(iii) complexes, **1b–4b**. The coordination geometry has a significant impact on the relative energies of the frontier orbitals (specifically, the d-orbital energies) and hence the relative energies of the singlet and triplet excited states, which subsequently affect the *k*_ISC_.

The two factors that determine the *k*_ISC_ are (1) the SOC matrix element <S_*n*_|*H*_SOC_|T_*m*_>, and (2) the energy gap (Δ*E*_ST_) between the coupling singlet (S_*n*_) and triplet (T_*m*_) excited states. The larger the *H*_SOC_ and the smaller the energy gap (Δ*E*_ST_), the faster will be *k*_ISC_. For effective SOC, this requires the metal d-orbitals of the coupling singlet and triplet excited states to have different orientations. For example, if S_*n*_ is derived from a metal-to-ligand charge transfer (MLCT) excited state where Au(d_*xz*_) orbital is involved, *H*_SOC_ would be zero if the triplet excited state is also an MLCT state that involves Au(d_*xz*_) orbital because of symmetry reasons.

**Fig. 11:**
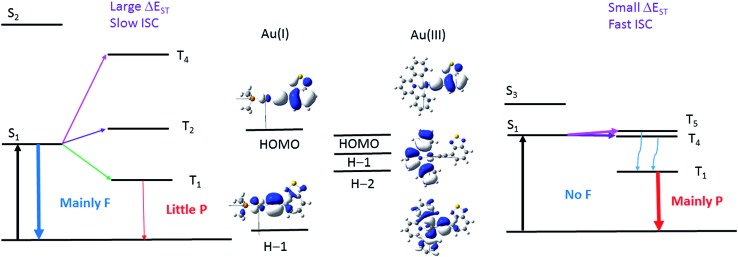
Illustration of the low-lying singlet and triplet excited states of Au(i) (left) and Au(iii) (right) complexes that accounts for the different photophysical behaviour of the Au(i) and Au(iii) complexes investigated in this work. S_1_ and T_1_ for both complexes are derived from HOMO → LUMO transitions; S_2_ and T_4_ excited states of **1a** are derived from ^1,3^[H–1 → LUMO] transitions while S_3_ and T_5_ excited states of **1b** are derived from ^1,3^[H–2 → LUMO] transitions. The d-orbitals involved in the T_2_ of **1a** and T_4_ of **1b** have the same orientations as their respective S_1_ excited state (see Tables S5, S6 and S9 in ESI†). The wavy blue arrows indicate internal conversion (IC) from the T_5_ to T_1_ excited state. F = fluorescence and P = phosphorescence.

The pair (**1a**, **1b**) has been chosen as a representative example to illustrate the different photophysical properties exhibited by the Au(i) and Au(iii) arylacetylide complexes studied in this work. From the DFT/TDDFT calculations, it is revealed that owing to the inherent linear coordination geometry of the Au(i) complex, the d-orbitals of the gold(i) ion is mainly destabilized by the arylacetylide ligand (Fig. 9 and Table S9 in ESI†). On the other hand, as Au(iii) complexes are assumed to have a square-planar four-coordinated geometry, thus, in addition to the antibonding interactions with the arylacetylide ligand, the d-orbitals of gold(iii) ion could also be destabilized by the cyclometalated [C^N^C] ligand (both π-type, *e.g.* H–1, and σ-type, *e.g.* H–2 in **1b**; Fig. 9 and Table S9 in ESI†); these latter interactions result in smaller d-orbital splittings in the Au(iii) series than the Au(i) series. In effect, S_1_ and S_2_ excited states are ∼4200 cm^–1^ apart for **1a** while the analogous splitting (between S_1_ and S_3_ excited states) is only ∼1300 cm^–1^ for **1b**. As S_2_ of **1a** is derived from ^1^[Au(d_*xy*_) → π*(C≡CR)]/^1^[π(C≡C) → π*(C≡CR)] (^1^MLCT/^1^ILCT) and S_3_ of **1b** from ^1^[Au(d_*xy*_) → π*(C≡CR)]/^1^[π(C≡C) → π*(C≡CR)]/^1^[π(C^N^C) → π*(C≡CR)] (^1^MLCT/^1^ILCT/^1^LLCT), *i.e.*, both are of charge-transfer type excited states, the singlet–triplet energy gaps for this type of transitions are small (Δ*E*(S_2_–T_4_) ∼ 1000 cm^–1^ for **1a** and Δ*E*(S_3_–T_5_) ∼ 900 cm^–1^ for **1b**) (T_4_ (**1a**) and T_5_ (**1b**) are the triplet counterpart of S_2_ (**1a**) and S_3_ (**1b**) respectively). As depicted in Fig. 11, the S_1_/T_5_ energy gap for **1b** is small but the S_1_/T_4_ energy gap for **1a** is large. In other words, the oxidation state of the gold ion affects the coordination geometry of the complex, which in turn change the interactions between the metal d-orbitals and ligand orbitals, giving rise to different d-orbital splitting and subsequently the singlet–triplet splitting (Δ*E*_ST_) of the two coupling excited states in the gold complexes.

Moreover, DFT/TDDFT calculations also revealed that there is a triplet excited state (T_4_) almost isoenergetic with the S_1_ excited state (<70 cm^–1^ below the S_1_ excited state) in **1b** such that even though the SOC between S_1_ and T_4_ is small due to the similar d-orbital orientations involved in both excited states, thermal energy could promote facile ISC. With **1a**, the closest triplet excited state (T_2_) to the S_1_ excited state is more than 500 cm^–1^ above the S_1_ excited state, which is more than twice the thermal energy at room temperature and SOC is also small between these two excited states as the d-orbitals involved are also of the same orientations. Thus, taken together both the SOC and Δ*E*_ST_, **1b** should have a much faster *k*_ISC_ than **1a**.

On the other hand, for the Bodipy-functionalized complexes, **4a** and **4b**, only ^1^ππ*(C≡CBodipy) fluorescence with no long-lived species are observed under ns-TRE and ns-TA measurements. The photophysical behavior of the Bodipy-functionalized complexes can be attributed to the intrinsically small band-gap of the Bodipy moiety. Due to the highly conjugated structure of Bodipy, the HOMO is much destabilized and there is a wide orbital energy gap between the HOMO and other occupied MOs, even in the case of **4b** which contains a [C^N^C] ligand. As a result, the HOMO/H–*x* orbital energy gap is the largest among the four arylacetylide ligands studied herein (H–*x* is the other occupied orbitals lower in energy than the HOMO; *x* = 1, 2, …). In effect, the closest T_*m*_ excited state that could have effective SOC with S_1_ is more than 1000 cm^–1^ above the S_1_ excited state. With such a large Δ*E*(S_1_–T_*m*_), thermal energy would be insufficient to promote ISC. Therefore, similar to the scenario in the case of **1a** (Fig. 11, left), ISC is sluggish for Au(i) and Au(iii) arylacetylide complexes bearing Bodipy.

### Mechanism for the generation of delayed fluorescence

**Fig. 12:**
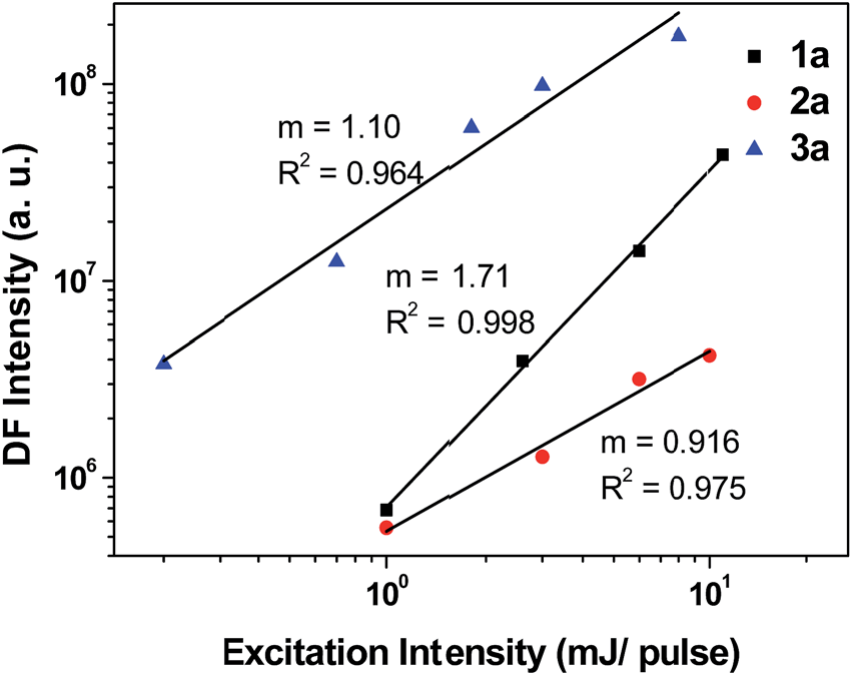
Dependence of delayed fluorescence of **1a–3a** in 5 × 10^–5^ M degassed CH_2_Cl_2_ with excitation power intensity. Emission intensity measured after a time delay of 1 μs. (Laser *λ*_exc_ = 355 nm; 0.2–11 mJ per pulse; diameter = 8 mm, integration time: 800 μs.)

From the ns-TRE measurements of **1a–3a**, DF contributes to the total fluorescence, though only a minute proportion (<3%, Table 3). In general, the mechanism of DF could be inferred from the dependence of the DF intensity (*I*_DF_) with the power of excitation light.^30^ According to Bässler, a quadratic dependence of excitation power with the DF intensity indicates that the mechanism of the DF is TTA with dominant phosphorescence.^30*b*^ On the other hand, a linear dependence of DF intensity with excitation power could be due to three possible mechanisms: TTA with dominant delayed-fluorescence, TADF, and GP-recombination. As depicted in Fig. 12, the plot of *I*_DF_ against excitation intensity in double-logarithm scale gave a slope of 1.71 ≈ 2 for **1a**; this nearly quadratic dependence is most consistent with the TTA mechanism with dominant phosphorescence. However, for **2a** and **3a** (slope = 0.916 and 1.10), both display nearly linear dependence between *I*_DF_ and excitation intensity. Therefore, it is not possible to confirm the mechanism for DF in the case of **2a** and **3a** by solely considering the excitation power dependence.

Time-dependence of *I*_DF_ and phosphorescence intensity (*I*_P_) could also give hints to the DF mechanism.^30,31^ For TTA with dominant DF, phosphorescence intensity decays with a power law, *I*_P_ ∝ *t* ^–1^ while *I*_DF_ is approximately constant at short time and *I*_DF_ ∝ *t* ^–2^ at longer time. For the GP-recombination mechanism, both DF and phosphorescence decay in accordance with the power law, *I*_DF_ ∝ *t* ^–1^, at both short and long times.^30*a*^ In the case of **2a** in 77 K glassy solution, both DF and phosphorescence decayed according to the power law: *I* ∝ *t* ^–1^ over the time intervals investigated (1 μs to 1.2 ms) (Fig. 6, inset), suggesting that the DF mechanism under this condition is most likely the GP-recombination mechanism. As for **3a**, there is no power law decay relation with both DF and phosphorescence and so it seems unlikely that GP-recombination is the mechanism for the generation of DF in **3a**. There is still not enough information to conclude on the DF mechanism for **3a**.

## Conclusion

A series of gold complexes with different oxidation states, gold(i) complexes [LAu(C≡CR)] and gold(iii) complexes [Au(C^N^C)(C≡CR)] bearing the same heterocyclic arylacetylides with narrow bandgap were synthesized and characterized. The photophysical behaviors with the gold ion in different oxidation states are strikingly different: fluorescence dominates the luminescence of the Au(i) complexes while phosphorescence takes over in the Au(iii) complexes. Detailed computational studies by DFT/TDDFT have accounted for these phenomena as a result of different coordination environments inherited from the gold ion in a particular oxidation state: a linear coordination geometry for Au(i) and a square-planar coordination geometry for Au(iii). This difference in coordination geometry subtly affects the energy separation between the coupling singlet and triplet excited states, leading to smaller Δ*E*_ST_ of the Au(iii) complexes than the Au(i) complexes and hence, larger *k*_ISC_ in the Au(iii) complexes than the Au(i) complexes. For the complexes bearing Bodipy-functionalized acetylide ligand, they only display prompt fluorescence. Computational analyses revealed that, due to the especially narrow bandgap of Bodipy, the Δ*E*_ST_ is still large even in the Au(iii) complex so that *k*_ISC_ could not compete with fluorescence radiative decay. Additionally, the mechanisms for the generation of DF in Au(i) complexes have been explored. To the best of our knowledge, this is the first report which systematically studies the effects of the metal ion oxidation state on the photophysical behaviours of transition-metal complexes.
