# Supplementary material for: The interplay between fluorescence and phosphorescence with luminescent gold(i) and gold(iii) complexes bearing heterocyclic arylacetylide ligands
Source: Chem Sci. 2016 Dec 5;8(3):2352–64. doi: 10.1039/c6sc03775e (PMC5365001; doi:10.1039/c6sc03775e)
Supplement: Supplementary file 1 [file SC-008-C6SC03775E-s001.pdf]

Electronic Supplementary Material (ESI) for Chemical Science.
This journal is © The Royal Society of Chemistry 2016

Electronic Supplementary Information for

The Interplay between Fluorescence and Phosphorescence in Luminescent Gold(I) and Gold(III) Complexes Bearing Heterocyclic Arylacetylide Ligands

Kaai Tung Chan[a], Glenna So Ming Tong[a], Wai-Pong To[a], Chen Yang[a], Lili Du[b], David Lee Phillips[b] and Chi-Ming Che*[a,c]*

a. *State Key Laboratory of Synthetic Chemistry, Institute of Molecular Functional Materials, Department of Chemistry, The University of Hong Kong, Pokfulam Road, Hong Kong SAR, China*
b. *Department of Chemistry, The University of Hong Kong, Hong Kong, China*
c. *Department of Chemistry, HKU Shenzhen Institute of Research and Innovation Shenzhen 518053, China*

Content

1. Experimental Details
   - 1.1. Chemical and Instrumentation
   - 1.2. Synthesis and Characterization
   - 1.3. X-ray Crystallographic data
   - 1.4. Cyclic voltammogram
2. Spectroscopic data
3. Computational Details
4. References

1. Experimental Details

1.1 Chemicals and Instrumentation

All chemicals were purchased unless specified. Reagent grade solvents were used for synthesis. For photophysical measurements and cyclic voltammetric measurements, HPLC grade solvents were used.

^{1}H, ^{13}C nuclear magnetic resonance (NMR) spectra were recorded on a Bruker DPX-300, Avance 400, DRX-500 or Avance 600 NMR spectrometer; ^{19}F and ^{31}P NMR were recorded on an Avance 400 NMR spectrometer; ^{11}B recorded on an Avance 600 NMR spectrometer. Solvents used for NMR measurements were all deuterated. The recorded chemical shifts (δ) for ^{1}H and ^{13}C in $CDCl_3$ were reported in ppm with reference to tetramethylsilane (TMS) (δ = 0). For other deuterated solvents, the chemical shifts were calibrated with the corresponding solvent residual peaks. ^{13}C, ^{19}F, ^{31}P and ^{11}B signals are all ^{1}H decoupled.

Mass spectra were recorded on a Finnigan MAT 95 mass spectrometer. Electron impact (EI) was used for characterizing organic compounds and fast-atom bombardment (FAB) was used for metal complexes. Elemental analyses were performed at the Institute of Chemistry, Chinese Acamdemy of Sciences, Beijing, China. Thermogravimetric analyses (TGA) were performed under nitrogen on a Perkin Elmer Pyris1 Thermogravimetric Analyzer; scan rate: 10°C/min; scan range: 40–800°C.

<u>Steady-state Photophysical Measurements</u>

Steady-state emission and excitation spectra were recorded on a Fluorolog 3 Spectrofluorometer. For measurements in solution at room temperature, samples were placed in a two-compartment cells consisting of a 10 mL-pyrex bulb and a

1–cm path length quartz curvette the solutions. The cells were sealed from the atmosphere with Rotaflo stopcocks. Solutions were degassed in a high-vacuum line for five times by freeze-thaw-pump cycles.

Luminescence quantum yields (Φ_s) were determined by the method of Demas and Crosby[1] using $[Ru(bpy)_3](PF_6)_2$ in degassed acetonitrile (Φ_r = 0.062), 9,10-bis(phenylethynyl)anthracence (BPEA) in degassed benzene (Φ_r = 0.85) or quinine sulfate in degassed 0.1 N H_2SO_4 (Φ = 0.546) as the standard. Φ_s were calculated by the following equation:

$$\Phi_s = \Phi_r \frac{B_r}{B_s} \left(\frac{n_s}{n_r}\right)^2 \left(\frac{D_s}{D_r}\right)$$

where the subscripts s and r denote the parameters for sample and reference respectively, n is the refractive index of solvents; D is the integrated emission intensity; B = 1 – 10^{-AL} (where A is the absorbance at the excitation wavelength, L is the optical path length in cm).

Solid samples or glassy solutions (EtOH:MeOH [4:1]; concentration: 10^{-6} to 10^{-5} M) were placed in 5-mm quartz tubes. For low-temperature 77K measurements, the quartz tubes containing the samples were placed in a quartz-walled Dewar filled with liquid-nitrogen.

Emission lifetimes (τ) were measured with a Quanta Ray DCR-3 pulsed Nd: YAG laser system (pulse λ_{exc} = 355 nm, pulse width: 8 ns). Emission decay signals were monitored as a function of time using a R928 photomultiplier tube. The lifetime values were estimated by fitting mono-exponential decay using Origin software.

Fluorescence lifetimes were measured by a time-correlated single photon counting (TCSPC) system using a NanoLED-375L pulsed laser diode with excitation wavelength at 371 nm (pulse width: < 200ps). Emission decay signals

were collected with a TBX picosecond photon detection module. Data collected were analyzed with the software DAS6.

Time-resolved Spectroscopy

Samples for nanosecond time-resolved emission or nanosecond transient absorption (ns-TA) were prepared in the same way as steady-state photophysical measurements. Measurements were performed on a LP920-KS Laser Flash Photolysis Spectrophotometer (Edinburgh Instruments Ltd). The excitation source was a Q-switched Nd:YAG laser system. Laser excitation wavelength (λ_{exc}) used was 355 nm (the third harmonic line). Pulse width was 10 ns with a repetition rate of 10 Hz. Emission signals monitored without time delay and within a gate width of 500 ns were assigned as prompt fluorescence (PF); those monitored with a time delay of 1 μs and within a gate width of 1 ms were assigned as delayed fluorescence (DF). Due to the weak delayed fluorescence intensity, the energy of the excitation source used was in the range of 0.2–11 mJ per pulse. For transient absorption measurement, the probe light source was a 450 W Xenon arc lamp. Signals were allowed to pass through a photomultiplier tube and recorded by the TDS 3012C digital signal analyzer. Data were processed with a PC-plugin controlled by the software L900.

Femtosecond time-resolved transient absorption (fs-TA) measurements were performed on a HELIOS setup equiped with a femtosecond regenerative amplified Ti:sapphire laser system (Spitfire Pro) in which the amplifier was seeded with the 120 fs laser pulses from an oscillator laser system (1k Hz). Details of the setup were reported previously.[2] The laser probe pulse was produced by utilizing about 100 mw of the amplified 800 nm laser pulses to generate a white-light continuum (430-750 nm) in a sapphire crystal and then this probe beam was split into two

parts before traversing the sample. One part of the probe laser beam goes through the sample while the other part of the probe laser beam goes to the reference spectrometer. For the present experiments, sample solutions were excited by a 400 nm pump beam (the second harmonic of the fundamental 800 nm, 50 mw) in a 2 mm path-length cuvette. The maximum time window is 3300 ps. Signals for each measurement were averaged for 1 s.

Femtosecond time-resolved emission (fs-TRE) measurements were performed on the same setup as fs-TA. The output 800 nm laser pulse (200 mw) is used as gate pulse while the 400 nm laser pulse (10 mw) (second harmonic) is used as the pump laser. After excitation by the pump laser, the sample fluorescence is focused into the nonlinear crystal (BBO) mixing with the gate pulse to generate the sum frequency signal. Broadband fluorescence spectra are obtained by changing the crystal angles and the spectra are detected by the air-cooled CCD.

Cyclic Voltammetry

Cyclic voltmmetric measurements were performed on a Princeton Applied Research electrochemical analyser (potentiostat/ galvanostat Model 273 A) in a three-compartment cell. Tetrabutylammonium hexafluorophosphate ($n\mathrm{Bu_4NPF_6}$, 0.1 M) in CH_2Cl_2, CH_3CN or DMF was used as the supporting electrolyte throughout this work. The solutions were degassed by Argon prior to measurements. Ag/$AgNO_3$ (0.1 M in CH_3CN), glassy carbon and platinum wire were used as the reference electrode, working electrode and counter electrode, respectively. All measurements were conducted at room temperature. Oxidation or reduction potential for reversible or quasi-reversible processes were reported as $E_{1/2}$ by taking the average of cathodic and anodic peak potentials. Peak potentials

were reported for irreversible redox process. The ferrocenium/ferrocene ($Cp_2Fe^{+/0}$) couple was used as the internal standard.

1.2 Synthesis and Characterization

General procedure for the synthesis of L1−L4

A mixture of iodo- or bromo- substituted aromatic ligand, $Pd(PPh_3)_2Cl_2$ (0.08 equiv.) and CuI (0.08 equiv.) was pump-filled under nitrogen, TMSA (3 equiv.) and triethylamine were added to the mixture under degassed condition. The dark solution was refluxed overnight. The product was purified by column chromatography on SiO_2 with hexane/dichloromethane as eluent. The (trimethysilyl)ethynyl ligand was deprotected by K_2CO_3 (1.2 equiv) followed by filtering through a silica plug or a SiO_2 column. The ethynyl ligands L1−L4 were obtained in 13% to 95% yields. The synthesis of L1–L4 and the precursors for these ligands are summarized in Scheme 1.

Synthesis of 2,1,3-benzothiadiazole: To 5 g o-phenylenediamine dissolved in 150 mL dichloromethane and triethylamine (4 equiv., 25 mL), thionyl chloride (2 equiv.) was added in dropwise at 0°C. The solution was heated to reflux for 5 h after which solvents were removed. Water was added and the solution was adjusted to pH 1 with conc. HCl. The product was purified by steam distillation. The pale yellow distillate was extracted with CH_2Cl_2 again. The product was obtained as off-white crystals. Yield: 4.52 g (71.8%)

4-Bromo-2,1,3-benzothiadiazole was obtained according to a reported procedure[3]. ^{1}H NMR (400 MHz, $CDCl_3$): δ 7.97 (d, 1H, J = 8.8 Hz), 7.84 (d, 1H, J = 7.8 Hz), 7.48 (t, 1H, J = 7.9 Hz).

4-Ethynyl-2,1,3-benzothiadiazole, L1 was synthesized according to the general procedure. Yield: 94.7%, light brown solid. MS (+EI) *m/z*: 160.0 [M+].

^1H NMR (400 MHz, $CDCl_3$): δ 8.03 (d, 1H, J = 8.8 Hz), 7.80 (d, 1H, J = 6.9 Hz), 7.56−7.60 (m, 1H), 3.59 (s, 1H, -C≡CH).

Synthesis of 3-bromo-7-(diethylamino)coumarin: 7-diethylaminocoumarin (1.06 g) was dissolved in acetic acid. Bromine (1 equiv.) was added in dropwise. Cream yellow precipitate formed immediately. The suspension was stirred for 3 h. The orange solids were filtered and washed with small amount of acetic acid and CH_2Cl_2. Yield: 1.04 g (72.5%), off-white solid. ^1H NMR (300 MHz, $CDCl_3$): δ 8.34 (s, 1H), 7.43 (d, 1H, J = 8.9 Hz), 6.71–6.75 (m, 1H), 6.55 (d, 1H, J = 2.4 Hz), 3.39–3.59 (m, 4H), 1.11 (t, 6H, J = 7.0 Hz).

3-Ethynyl-7-(diethylamino)coumarin, L2 was synthesized according to the general procedure. Yield: 13.0%, orange powder. MS (+EI) *m/z*: 241.1 [M+]. ^1H NMR (300 MHz, [D_6]DMSO): δ 8.18 (s, 1H), 7.43 (d, 1H, J = 8.9 Hz), 6.73 (dd, 1H, J = 8.9 Hz and 2.2 Hz), 6.53 (d, 1H, J = 2.1 Hz), 4.24 (s, 1H, -C≡CH), 3.43−3.50 (m, 4H, masked by H_2O), 1.12 (t, 6H, J = 7.0 Hz).

Synthesis of 4-bromo-N-butylnaphthalimide: a mixture of 4-bromo-1,8-napthalic anhydride (1 g) and N-butylamine (1.2 equiv.) were suspended in ethanol. The solution was refluxed overnight. The solids were filtered and washed with ethanol. Yield: 0.59g (47.4%), light brownish solid. ^1H NMR (300 MHz, $CDCl_3$): δ 8.66 (d, 1H, J = 7.1 Hz), 8.57 (d, 1H, J = 8.2 Hz), 8.42 (d, 1H, J = 8.0 Hz), 8.04 (d, 1H, J = 7.8 Hz), 7.85 (t, 1H, J = 7.9 Hz), 4.17 (m, 2H), 1.41–1.48 (m, 2H), 1.22–1.26 (m, 2H), 0.98 (t, 3H, J = 7.1 Hz).

4-Ethynyl-N-butylnaphthalimide, L3 was synthesized according to the

general procedure. Yield: 48.1%, white solid. MS (+EI) *m/z*: 227.1 [M+]. ^{1}H NMR (300 MHz, $CDCl_3$): δ 8.65 (t, 2H, *J* = 8.2 Hz), 8.54 (d, 1H, *J* = 7.6 Hz), 7.94 (d, 1H, *J* = 7.6 Hz), 7.83 (t, 1H, *J* = 7.8 Hz), 4.18 (t, 2H, *J* = 7.6 Hz), 3.73 (s, 1H, -C≡CH), 1.70−1.73 (m, 2H), 1.42−1.48 (m, 2H), 0.98 (t, 3H, *J* = 7.3 Hz).

Synthesis of Bodipy: Under nitrogen atmosphere, benzoyl chloride (1.4 g, 0.55 equiv.) was added in dropwise to 2,4-dimethylpyrrole (2 mL) in CH_2Cl_2. The reddish orange mixture was stirred at RT. After stirring overnight, the solution becomes dark red. Triethylamine (10 mL) was added, followed by the addition of $BF_3{\cdot}OEt_2$ (10 mL) at 0°C. The dark brown solution was allowed to stir for another 3 h. After which saturated Na_2CO_3 solution was added to quench the remaining acid. The crude product was extracted with CH_2Cl_2/H_2O. Subsequent column chromatography in SiO_2 with hexane:CH_2Cl_2 (1:1) yielded the desired orange powder. Yield: 0.59 g (20.0%). ^{1}H NMR (300 MHz, $CDCl_3$): δ 7.47 (m, 3H, br), 7.26 (m, 2H, br), 5.97 (s, 2H), 2.55 (s, 6H), 1.37 (s, 6H).

Synthesis of Iodo-Bodipy: 0.59 g Bodipy was dissolved in 60 mL CH_2Cl_2. N-iodosuccinimide (1 equiv.) dissolved in 30 mL CH_2Cl_2 was added dropwise. After stirring for 1 h, solvents were removed. The crude was purified by SiO_2 column chromatography. Yield: 0.45 g (54.9%), purplish red solid. ^{1}H NMR (300 MHz, $CDCl_3$): δ 7.49–7.50 (m, 3H), 7.26 (m, 2H), 6.04 (s, 1H), 2.63 (s, 3H), 2.57 (s, 3H), 1.38 (s, 6H).

H–C≡CBodipy, L4 was synthesized according to the general procedure. Yield: 66.6%, orange-red powder. MS (+EI) *m/z*: 348.2 [M+]. ^{1}H NMR (300 MHz, $CDCl_3$): δ 7.49−7.50 (m, 3H), 7.26−7.28 (m, 2H), 6.04 (s, 1H), 3.28 (s, 1H, -

C≡CH), 2.64 (s, 3H), 2.58 (s, 3H), 1.44 (s, 3H), 1.39 (s, 3H).

Scheme S1 Syntheses of L1–L4 and their precursors.

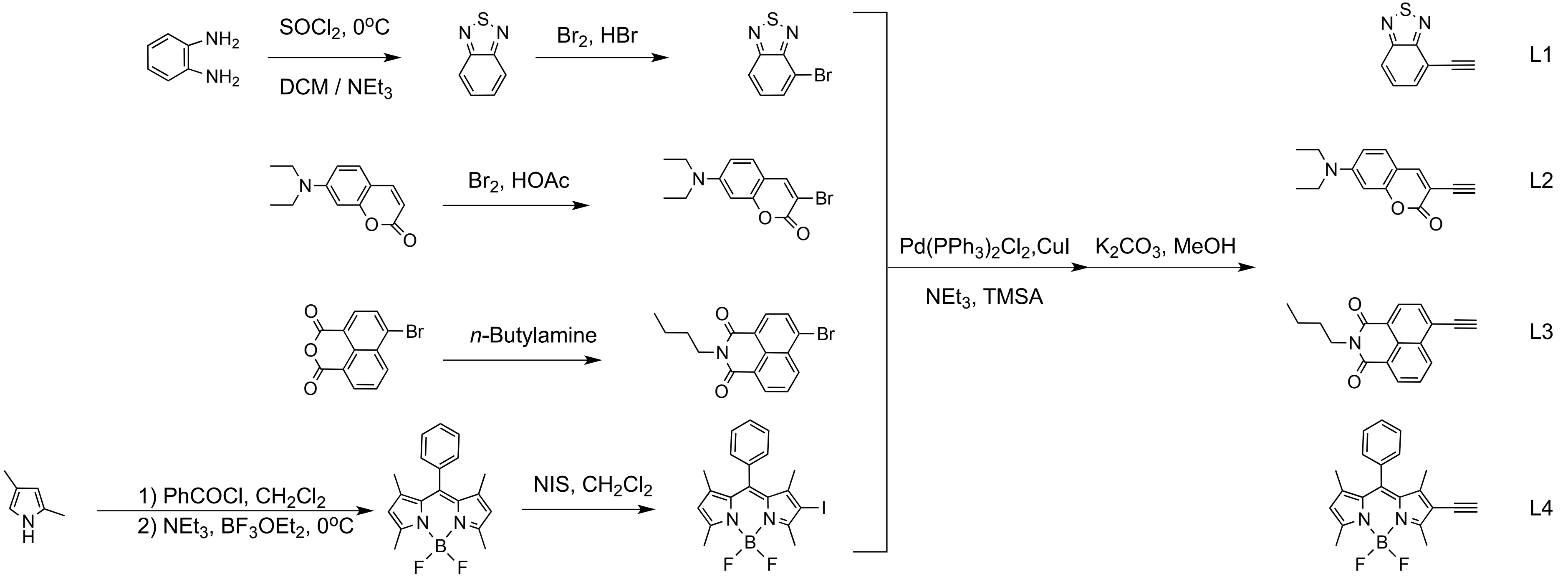

Synthesis of the Au(I) precursor complexes

Au(tht)Cl: $KAuCl_4$ (490 mg, 1.3 mmol) was dissolved in MeOH. Excess tetrahydrothiophene (tht) was added dropwise to the solution while white precipitates were forming. The addition was ceased until the yellow tint of the suspension had disappeared. The suspension was allowed to stir for another hour after which products were filtered and washed with MeOH. Yield: White powder. 410 mg (98.7%).

(Cy_3P)AuCl: To a solution of Au(tht)Cl (415 mg, 1.3 mmol.) dissolved in CH_2Cl_2 (30 mL), tricyclohexylphosphine (359 mg, 1.3 mmol) was added. The clear solution was stirred at room temperature overnight. After filtering through celite, the solution was concentrated. The products were precipitated and washed with MeOH. Yield: 439 mg (66.0%).

(RNC)AuCl (RNC = 2,6-dimethylphenylisocyanide): It was synthesized similar to (Cy_3P)AuCl by using 2,6-dimethylphenyl isocyanide (52 mg, 1.1 equiv.) instead of tricyclohexylphosphine. The crude product was washed with hexane to afford white powders. Yield: 95 mg (72.2%). ^1H NMR (300 MHz, $CDCl_3$): δ 7.35 (t, 1H, J = 7.6 Hz), 7.18 (d, 2H, J = 7.7 Hz), 2.44 (s, 6H).

(NHC)AuCl (NHC = 1,3-dimethylimidazol-2-ylidene): A mixture of 1,3-dimethylimidazolium iodide (50 mg, 0.22 mmol) and silver(I) oxide (26 mg, 0.11 mmol) were stirred in acetonitrile overnight. The grey suspension became white overnight. The crude was filtered through celite. Solvent was removed and the silver imidazolium salt was precipitated from ether (Yield: 57 mg, 78%). The

silver salt (57 mg, 0.086 mmol) obtained was stirred with Au(tht)Cl (55 mg, 0.17 mmol) in CH_2Cl_2 overnight. After reaction overnight, the crude was filtered through celite, followed by concentration and precipitation by ether. The product was obtained as white solid. Yield: White powder. 32 mg (56.6%). ^{1}H NMR (300 MHz, $CDCl_3$): δ 6.93 (s, 2H), 3.83 (s, 6H).

General procedure for the synthesis of **1a**−**6a**

Ethynyl ligands L1−L4 were stirred with sodium methoxide (2 equiv.) in CH_3OH. (Cy_3P)AuCl (**1a**–**4a**) or (RNC)AuCl (**5a**) or (NHC)AuCl (**6a**) (1 equiv.) dissolved in small amount of CH_2Cl_2 was added subsequently. After stirring at RT overnight, solvents are removed. The product was extracted with CH_2Cl_2/H_2O. Precipitation of a concentrated CH_2Cl_2 solution with MeOH followed by recrystallization in CH_2Cl_2 / hexane gave the pure complexes.

1a: Yellow crystal. Yield: 17.8 mg (71.6%). MS (+FAB) *m/z*: 637.0 [M+]. ^{1}H NMR (400 MHz, $CDCl_3$): δ 7.85 (d, 1H, J = 8.7 Hz), 7.72 (d, 1H, J = 7.2 Hz), 7.46−7.50 (m, ^{1}H), 1.26−2.03 (m, 33H, Cy); ^{31}P{1H} NMR ($CDCl_3$): δ 56.1; ^{13}C{^{1}H} NMR (100 MHz, $CDCl_3$): δ 155.7, 154.7, 146.2 (d, $^2J_{CP}$ = 130.7 Hz, Au–C≡C), 138.3, 132.3, 129.7, 119.9, 98.4 (d, $^3J_{CP}$ = 24.4 Hz, Au−C≡C), 33.3 (d, $^1J_{CP}$ = 27.7 Hz, Cy), 30.8 (s, Cy), 27.3 (d, $^2J_{CP}$ = 11.6 Hz, Cy), 26.0 (s, Cy). Elemental analyses calcd for $C_{26}H_{36}AuN_2PS·0.2CH_2Cl_2$: C, 48.15; H, 5.61; N, 4.29. Found: C, 48.16; H, 5.58; N, 4.21.

2a: Light orange powder. Yield: 22 mg (78.7%). MS (+FAB) *m/z*: 717.0 [M+]. ^{1}H NMR (400 MHz, $CDCl_3$): δ 7.70 (s, 1H), 7.16 (d, 1H, J = 8.8 Hz), 6.52

(dd, 1H, J = 8.8 Hz and 2.5 Hz), 6.45 (d, 1H, J = 2.3 Hz), 3.38−3.41 (m, 4H), 1.24−2.00 (m, 33H, Cy), 1.19 (t, 6H, J = 7.08 Hz); $^{31}P\{^{1}H\}$ NMR ($CDCl_3$): δ 56.2; $^{13}C\{^{1}H\}$ NMR (100 MHz, $CDCl_3$): δ 162.1, 155.6, 150.2, 144.2, 143.0 (d, $^{2}J_{CP}$ = 131.0 Hz, Au−C≡C), 138.2, 128.3, 109.0, 108.9, 107.2, 97.4 (d, $^{3}J_{CP}$ = 19.9 Hz, Au−C≡C), 44.9, 33.3 (d, $^{1}J_{CP}$ = 27.7 Hz, Cy), 30.8 (s, Cy), 27.2 (d, $^{2}J_{CP}$ = 11.8 Hz, Cy), 26.0 (s, Cy), 12.6. Elemental analyses calcd for $C_{33}H_{47}AuNO_2P$: C, 55.23; H, 6.60; N, 1.95. Found: C, 55.62; H, 6.89; N, 2.01.

3a: Yellow solid. Yield: 26 mg (70.7%). MS (+FAB) *m/z*: 754.0 [M+]. ^{1}H NMR (400 MHz, $CDCl_3$): δ 8.93 (d, 1H, J = 8.2 Hz), 8.58 (d, 1H, J = 6.7 Hz), 8.46 (d, 1H, J = 7.7 Hz), 7.87 (d, 1H, J = 7.7 Hz), 7.74 (t, 1H, J = 7.8 Hz), 4.16 (t, 2H, J = 7.5 Hz), 1.26−2.08 (m, 33H, Cy), 1.69−1.72 (m , 2H), 1.43-1.47 (m, 2H), 0.97 (t, 3H, J = 7.3 Hz); $^{31}P\{^{1}H\}$ NMR ($CDCl_3$): δ 56.3; $^{13}C\{^{1}H\}$ NMR (100 MHz, $CDCl_3$): δ 162.1, 155.6, 150.2, 144.2, 142.9 (d, $^{2}J_{CP}$ = 130.5 Hz, Au−C≡C), 138.2, 128.3, 109.0, 108.9, 107.2, 97.5 (d, $^{3}J_{CP}$ = 24.4 Hz, Au−C≡C), 44.9, 33.3 (d, $^{1}J_{CP}$ = 27.8 Hz, Cy), 30.8 (s, Cy), 27.2 (d, $^{2}J_{CP}$ = 11.3 Hz, Cy), 26.0 (s, Cy), 12.6. Elemental analyses calcd for $C_{36}H_{47}AuNO_2P{\cdot}0.5H_2O$: C, 56.69; H, 6.34; N, 1.84. Found: C, 56.88; H, 6.46; N, 1.80.

4a: Purple crystal. Yield: 24 mg (76.6%). MS (+FAB) *m/z*: 824.0 [M+]. ^{1}H NMR (300 MHz, CD_2Cl_2): δ 7.50−7.53 (m, 3H), 7.28−7.31 (m, 2H), 6.00 (s, 1H), 2.62 (s, 3H), 2.51 (s, 3H), 1.27−2.07 (m, 33H, Cy), 1.38 (s, 3H), 1.46 (s, 3H). $^{31}P\{^{1}H\}$ NMR ($CDCl_3$): δ 56.1; $^{19}F\{^{1}H\}$ NMR ($CDCl_3$): δ –146.5 (dd, J = 33.9 Hz and 65.9 Hz, BF_2); $^{11}B\{^{1}H\}$ NMR (160 MHz, $CDCl_3$): δ 3.76; $^{13}C\{^{1}H\}$ NMR (150 MHz, $CDCl_3$): δ 159.2, 154.7, 144.7, 143.8, 143.5, 142.5, 141.3, 135.1, 131.2 (d,

$^2J_{CP}$ = 123.5 Hz, Au–C≡C), 129.1, 128.8, 128.0, 120.8, 118.4, 94.6 (d, $^3J_{CP}$ = 24.9 Hz, Au−C≡C), 33.2 (d, $^1J_{CP}$ = 27.6 Hz, Cy), 30.7, 27.1 (d, $^2J_{CP}$ = 11.6 Hz, Cy), 25.9, 14.5, 14.3, 13.9, 13.6. Elemental analyses calcd for $C_{39}H_{51}AuBF_2N_2P{\cdot}0.5H_2O$: C, 56.19; H, 6.29; N, 3.36. Found: C, 56.19; H, 6.18; N, 3.35.

5a: Orange powder. Yield: 25 mg (53.2%). MS (+FAB) *m/z*: 487.9 [M+]. ^{1}H NMR (400 MHz, $CDCl_3$): δ 7.87 (d, 1H, *J* = 8.7 Hz), 7.71 (d, 1H, *J* = 6.9 Hz), 7.51 (dd, 1H, *J* = 8.7 and 7.0 Hz), 7.34 (t, 1H, *J* = 7.7 Hz), 7.17 (d, 2H, *J* = 7.7 Hz), 2.45 (s, 6H). ^{13}C{^{1}H} NMR (150 MHz, $CDCl_3$): δ 155.8, 154.6, 136.3, 132.6, 131.2, 131.2, 131.1, 129.6, 128.6, 128.5, 120.5, 118.6, 99.0 (Au−C≡C), 18.8 (-CH_3). Elemental analyses calcd for $C_{17}H_{12}AuN_3S$: C, 41.90; H, 2.48; N, 8.62. Found: C, 41.58; H, 2.08; N, 8.42.

6a: Light orange powder. Yield: 19 mg (68.8%). MS (+FAB) *m/z*: 452.8 [M+]. ^{1}H NMR (400 MHz, $CDCl_3$): δ 7.83 (d, 1H, *J* = 8.8 Hz), 7.71 (d, 1H, *J* = 7.0 Hz), 7.49 (t, 1H, *J* = 7.9 Hz), 6.91 (s, 2H), 3.87 (s, 6H). ^{13}C{^{1}H} NMR (150 MHz, $CDCl_3$): δ 187.7 (Au–C_{sp2}), 155.9, 154.7, 138.5, 132.1, 129.7, 121.8, 119.7, 119.7, 100.2 (Au−C≡C), 38.0 (N–CH_3). Elemental analyses calcd for $C_{13}H_{11}AuN_4S$: C, 34.52; H, 2.45; N, 12.39. Found: C, 34.23; H, 2.13; N, 12.19.

General procedure for the synthesis of **1b−4b**

A mixture of [Au(C^N^C)Cl] (40 mg), ethynyl ligand L1–L4 (1.05 equiv.), triethylamine (1–2 mL), copper(I) iodide (2 mg) was stirred in dichloromethane (20 mL) for 3 hours under argon. The solvent was then evaporated under reduced

pressure. The crude product was purified by column chromatography on SiO_2 using a mixture of hexane and dichloromethane as eluent.

1b: Off-white solid. Yield: 13 mg (50.8%). MS (+FAB) *m/z*: 585.9 [M+]. ^{1}H NMR (400 MHz, $CDCl_3$): δ 8.24 (d, 2H, *J* = 7.1 Hz), 7.95 (d, 1H, *J* = 8.7 Hz), 7.89 (t, 1H, *J* = 8.0 Hz), 7.84 (d, 1H, *J* = 6.8 Hz), 7.57−7.60 (m, 3H), 7.51 (d, 2H, *J* = 8.0 Hz), 7.42 (t, 2H, *J* = 7.4 Hz), 7.27−7.31 (m, 2H). Elemental analyses calcd for $C_{25}H_{14}AuN_3S·0.5H_2O$: C, 50.51; H, 2.54; N, 7.07. Found: C, 50.20; H, 2.14; N, 6.95.

2b: Yellow solid. Yield: 48 mg (84.0%). MS (+FAB) *m/z*: 669.2 [M+]. ^{1}H NMR (400 MHz, [D_6]DMSO): δ 8.18 (t, 1H, *J* = 8.0 Hz), 8.10 (s, 1H), 8.00 (t, 4H, *J* = 7.7 Hz), 7.92 (d, 2H, *J* = 7.5 Hz), 7.48 (d, 1H, *J* = 8.9 Hz), 7.43 (t, 2H, *J* = 7.3 Hz), 7.34 (d, 1H, *J* = 7.3 Hz), 6.73 (dd, 1H, *J* = 8.9 Hz and 2.3 Hz), 6.56 (d, 1H, *J* = 2.2 Hz), 3.44−3.48 (m, 4H), 1.14 (t, 6H, *J* = 7.0 Hz); $^{13}C\{^1H\}$ NMR (125 MHz, $CDCl_3$): δ 166.1, 163.9, 160.5, 155.5, 150.5, 149.4, 144.7, 143.8, 136.1, 131.6, 129.1, 127.1, 126.0, 118.2, 109.2, 108.1, 106.2, 96.7, 96.5, 95.9, 44.1, 12.4. Elemental analyses calcd for $C_{32}H_{25}AuN_2O_2$: C, 57.66; H, 3.78; N, 4.20. Found: C, 57.43; H, 3.79; N, 4.32.

3b: Yellow solid. Yield: 34 mg (55.9%). MS (+FAB) *m/z*: 702.8 [M+]. ^{1}H NMR (400 MHz, [D_6]DMSO): δ 8.84 (d, 1H, *J* = 6.3 Hz), 8.50 (d, 1H, *J* = 5.4 Hz), 8.17 (t, 1H, *J* = 6.4 Hz), 8.17 (t, 1H, *J* = 6.4 Hz), 7.95−8.00 (m, 4H), 7.89−7.92 (m, 4H), 7.40 (t, 2H, *J* = 5.6 Hz), 7.33 (t, 2H, *J* = 5.3 Hz), 4.04 (t, 2H, *J* = 5.3 Hz), 1.61−1.62 (m, 2H), 1.35−1.36 (m, 2H), 0.93 (t, 3H, *J* = 5.9 Hz). Elemental

analyses calcd for $C_{35}H_{25}AuN_2O_2$: C, 59.84; H, 3.59; N, 3.99. Found: C, 59.75; H, 3.63; N, 4.11.

4b: Purplish red solid. Yield: 51 mg (79.8%). MS (+FAB) *m/z*: 772.8 [M+]. ^{1}H NMR (400 MHz, [D$_6$]DMSO): δ 8.16 (t, 1H, J = 8.0 Hz), 7.98 (d, 2H, J = 8.1 Hz), 7.90 (d, 2H, J = 7.7 Hz), 7.86 (d, 2H, J = 7.2 Hz), 7.59−7.61 (m, 3H), 7.43−7.45 (m, 2H), 7.39 (t, 2H, J = 7.4 Hz), 7.31 (t, 2H, J = 7.5 Hz), 6.25 (s, 1H), 2.68 (s, 3H), 1.37 (s, 3H); ^{13}C{^{1}H} NMR (100 MHz, CDCl$_3$): δ 166.9, 165.0, 158.1, 155.5, 149.0, 143.1, 143.0, 142.1, 141.5, 136.5, 135.1, 132.0, 129.2, 129.0, 128.1, 126.7, 125.2, 121.2, 116.7, 98.2, 92.3, 14.6, 14.4, 13.9, 13.6; ^{11}B{^{1}H} NMR (160 MHz, CDCl$_3$): δ 3.83 (t, $^1J_{BF}$ = 40.6 Hz). Elemental analyses calcd for $C_{38}H_{29}AuBF_2N_3 \cdot 0.5H_2O$: C, 58.33; H, 3.86; N, 5.37. Found: C, 58.15; H, 3.83; N, 5.41.

1.3 X-ray Crystallographic data

(a)

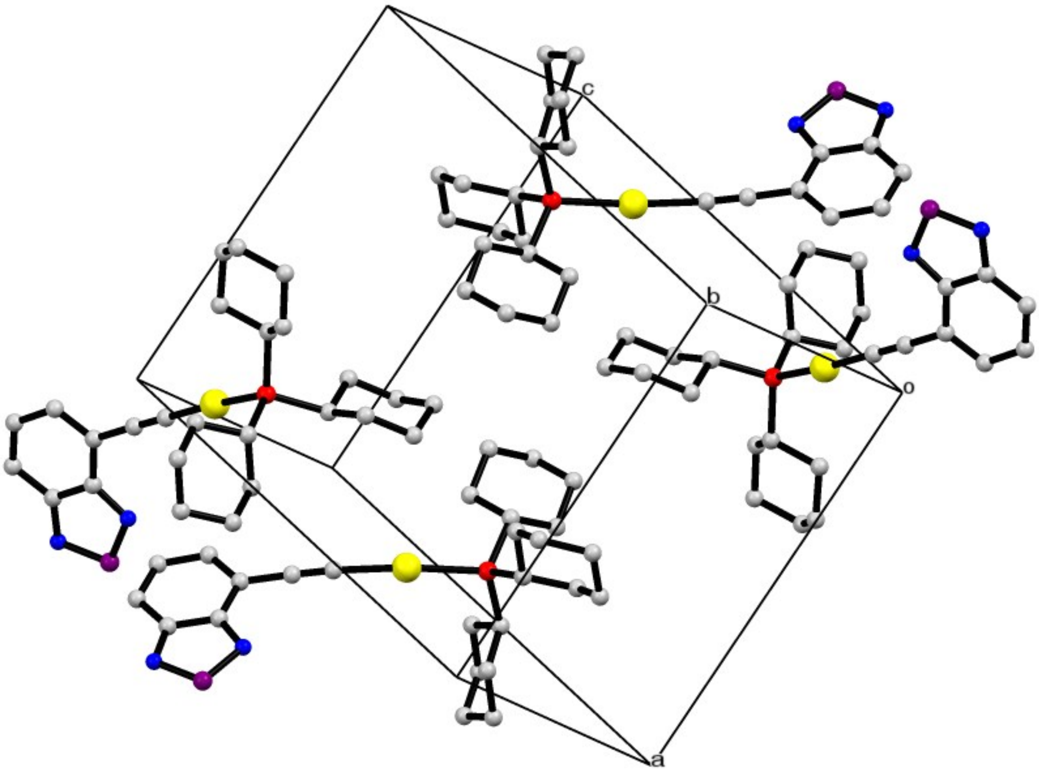

(b)

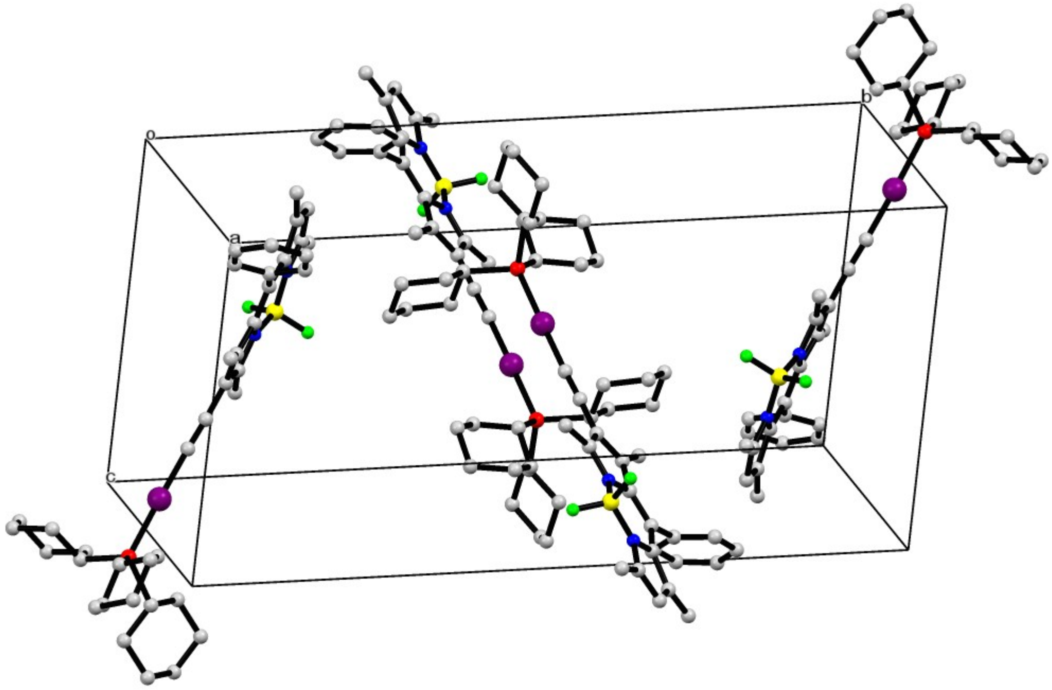

Figure S1 Crystal packing diagrams of individual molecules of (a) **1a** (grey: C; red: P; yellow: Au; blue: N; and purple: S) and (b) **4a** (grey: C; red: P; yellow: B; green: F; blue: N; and purple: Au) in a unit cell.

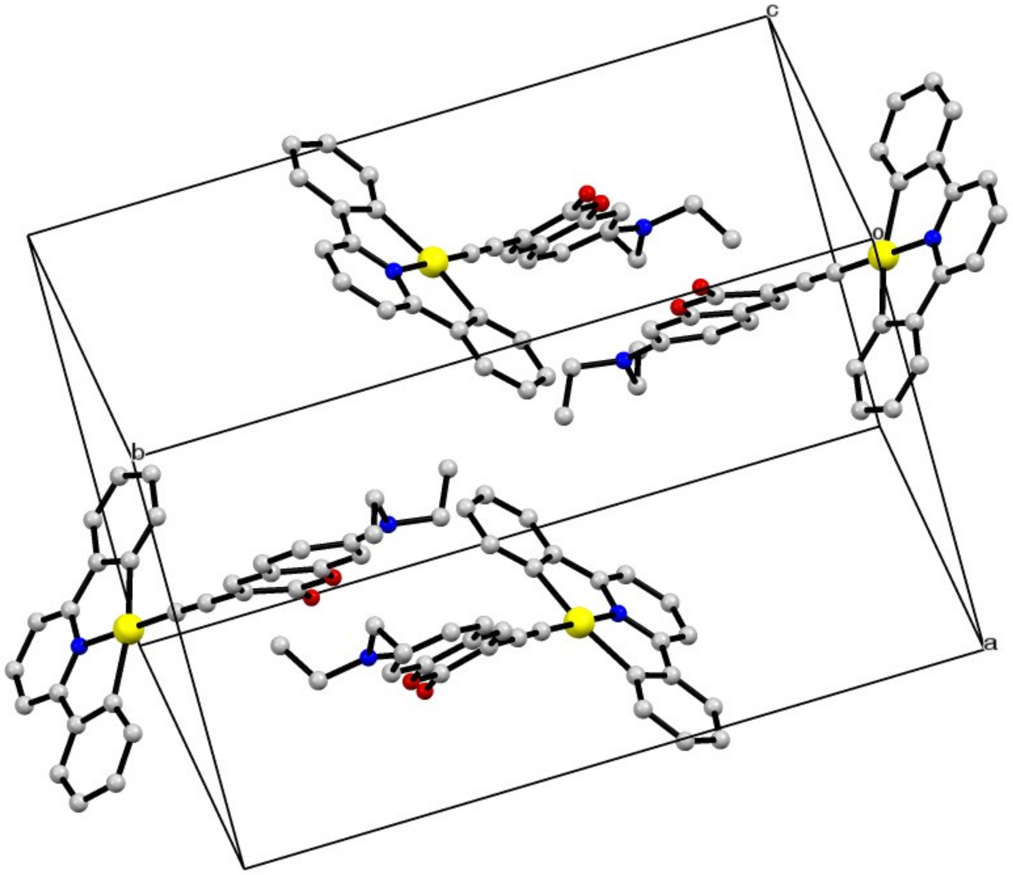

Figure S2 Crystal packing diagram of individual molecules of **2b** (grey: C; red: O; yellow: Au; and blue: N) in a unit cell.

Table S1 Crystal data of **1a**, **4a** and **2b**

Complex	**1a**	**4a**	**2b**
Empirical formula	$C_{26}H_{38}AuN_2PS$	$C_{39}H_{51}AuBF_2N_2P$	$C_{32}H_{25}AuN_2O_2$
Formula weight	638.58	824.56	666.51
Temperature/K	100	100	100
Crystal system	monoclinic	monoclinic	monoclinic
Space group	$P2_1/c$	$P2_1/n$	$P2_1/c$
a/Å	12.4327(5)	10.9501(6)	11.4778(17)
b/Å	15.7628(7)	25.1526(13)	20.461(3)
c/Å	12.9117(6)	13.6349(7)	11.6734(17)
α/°	90	90	90
β/°	97.2061(9)	102.5560(10)	111.062(2)
γ/°	90	90	90
Volume/Å^3	2510.37(19)	3665.6(3)	2558.3(7)

Z	4	4	4
ρ_{calc}g/cm^3	1.69	1.494	1.73
μ/mm^{-1}	12.501	8.261	11.062
F(000)	1272	1664.0	1304
Crystal size/mm^3	0.3 × 0.2 × 0.1	0.04 × 0.04 × 0.03	0.3 × 0.2 × 0.2
Radiation (λ = 1.54178)	Cu Kα	Cu Kα	Cu Kα
2θ range for data collection/°	7.166 to 135.01	7.028 to 135.054	8.254 to 135.27
Index ranges	-14 ≤ h ≤ 14, -18 ≤ k ≤ 18, -15 ≤ l ≤ 15	–13 ≤ h ≤ 11, –26 ≤ k ≤ 29, –16 ≤ l ≤ 13	-13 ≤ h ≤ 13, -24 ≤ k ≤ 24, -13 ≤ l ≤ 13
Reflections collected	27779	51256	38228
Independent reflections	4420 [R_{int} = 0.0521, R_{sigma} = 0.0309]	6512 [R_{int} = 0.0657, R_{sigma} = 0.0388]	4539 [R_{int} = 0.0378, R_{sigma} = 0.0198]
Data/restraints/parameters	4420/180/250	6512/488/474	4539/0/337
Goodness–of–fit on F^2	1.132	1.034	1.14
Final R indexes [I>=2σ (I)]	R_1 = 0.0317, wR_2 = 0.0853	R_1 = 0.0409, wR_2 = 0.1069	R_1 = 0.0240, wR_2 = 0.0645
Final R indexes [all data]	R_1 = 0.0322, wR_2 = 0.0859	R_1 = 0.0417, wR_2 = 0.1077	R_1 = 0.0241, wR_2 = 0.0645
Largest diff. peak/hole / e Å^{-3}	1.19/-2.40	1.18/–3.73	0.95/-0.65

[a] $R_1 = \sum||F_o| - |F_c|| / \sum|F_o|$; [b] $wR_2 = [\sum w(|F_o| - |F_c|)^2 / \sum w|F_o|^2]^{1/2}$

Table S2 Selected bond distances (Å) and angles (°) of **1a**

Crystal **1a**			
Au1–C19	2.049(5)	P1–Au1–C1	175.00(13)
C19–C20	1.146(7)	Au1–C19–C20	170.9(4)
C20–C21	1.444(6)	C19–C20–C21	178.1(5)
Au1–P1	2.2852(10)	C1–P1–Au1	113.83(15)
P1–C1	1.849(4)	C7–P1–Au1	109.24(13)

P1–C7	1.837(4)	C13–P1–Au1	110.60(13)
P1–C13	1.844(4)		
C25–N1	1.345(7)		
N1–S1	1.607(4)		
C25–C26	1.430(7)		

Table S3 Selected bond distances (Å) and angles (°) of **4a**

Crystal **4a**			
Au1–C1	2.001(5)	P1–Au1–C1	178.1(2)
Au1–P1	2.2713(11)	Au1–C1–C2	177.6(5)
C1–C2	1.191(8)	C1–C2–C3	177.7(6)
C2–C3	1.429(7)	C22–P1–Au1	112.92(14)
N1–B1	1.552(6)	C28–P1–Au1	110.46(15)
N2–B1	1.545(6)	C8–N1–B1	125.8(4)
B1–F1	1.389(5)	C10–N2–B1	125.5(3)
B1–F2	1.386(6)	N1–B1–F1	109.7(3)
P1–C22	1.834(4)	N2–B1–F2	110.5(3)
P1–C28	1.841(5)		

Table S4 Selected bond distances (Å) and angles (°) of **2b**

Crystal **2b**			
Au1–C1	2.068(3)	C1–Au–N1	81.19(12)
Au1–N1	2.006(2)	C17–Au1–N1	81.28(13)
Au1–C17	2.073(4)	C1–Au1–C17	162.44(14)
Au1–C18	1.969(4)	N1–Au1–C18	179.47(13)
C18–C19	1.197(5)	Au1–C18–C19	178.3(3)
C19–C20	1.431(5)	C18–C19–C20	178.3(4)
N2–C26	1.364(4)		

1.4 Cyclic voltammogram

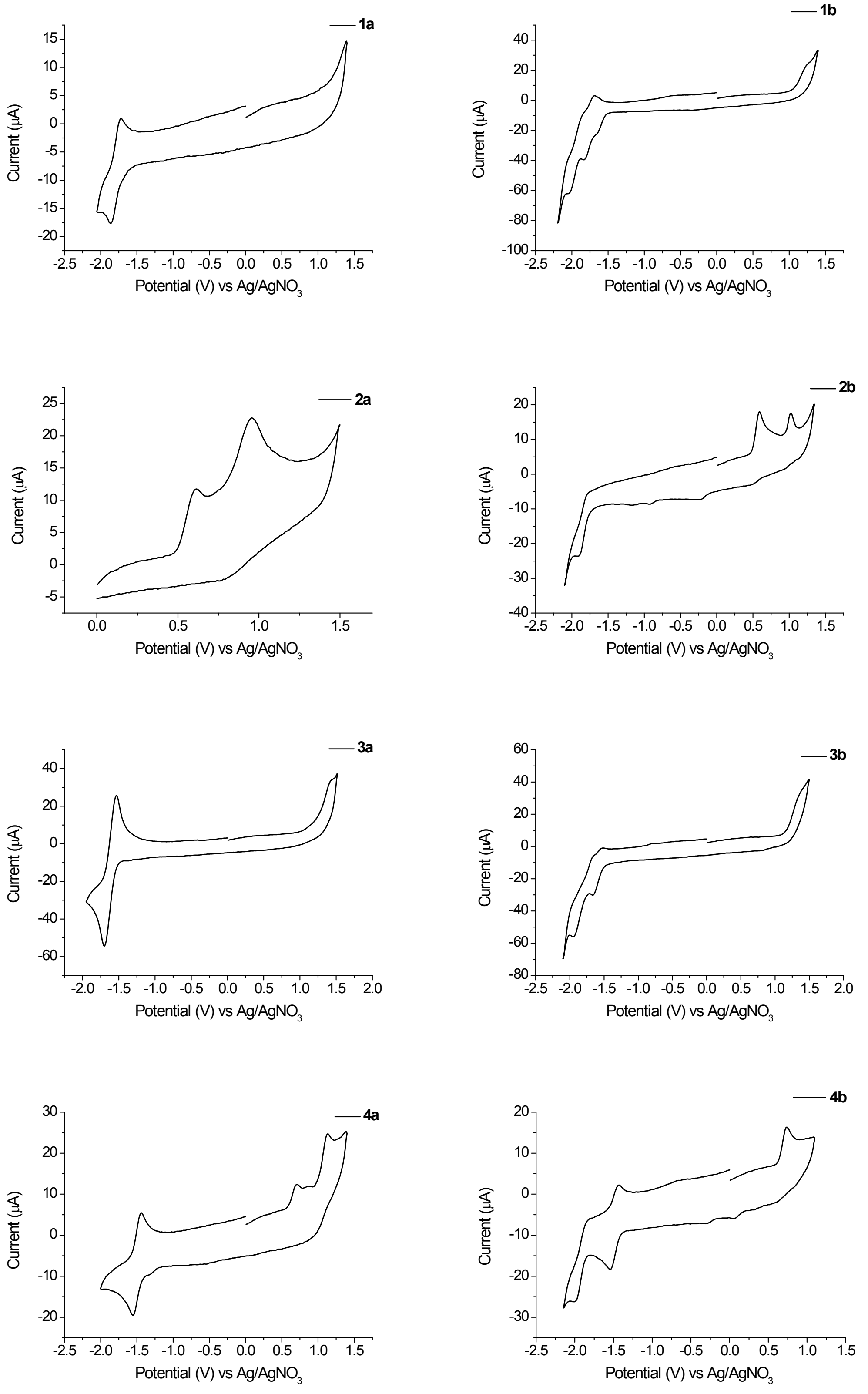

Figure S3 Cyclic voltammograms of (left) **1a–4a** and (right) **1b–4b** in CH_2Cl_2 with 0.1 M nBu_4NPF_6 as the supporting electrolyte at 298 K. ($E_{1/2}$ of $Cp_2Fe^{+/0}$

occurs at +0.15–0.16 V)

2. Spectroscopic data

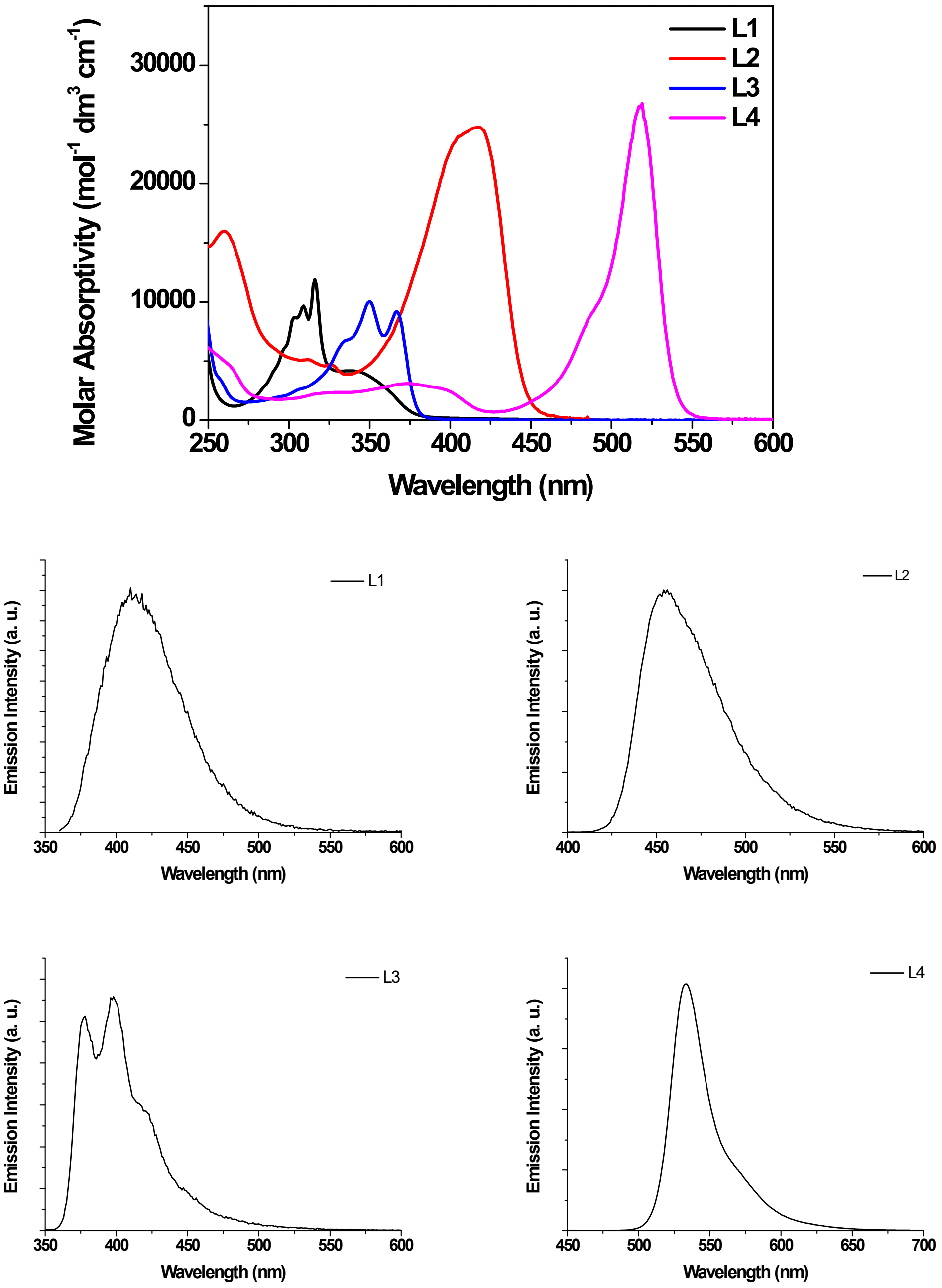

Figure S4 UV-vis absorption and emission spectra of L1–L4 in CH_2Cl_2 at 298 K (2×10^{-5} M).

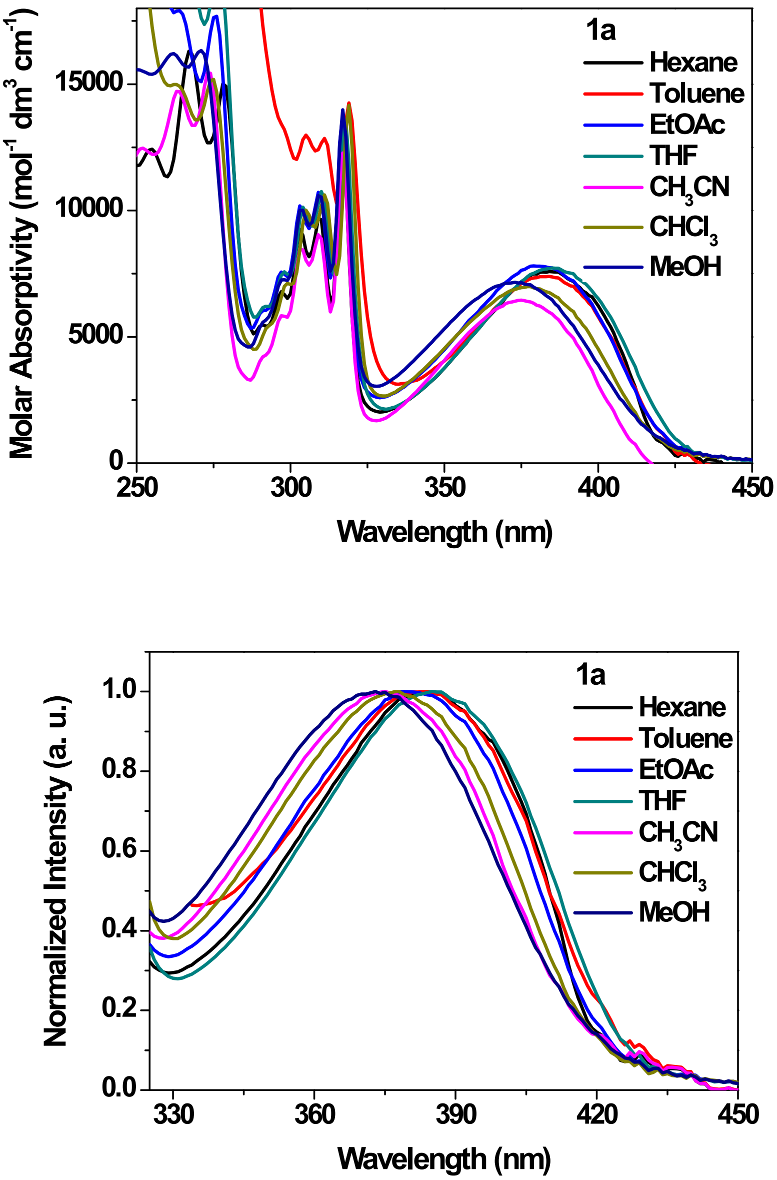

Figure S5 UV-vis absorption spectra (top) and the normalized absorption spectra (bottom) of **1a** at 325–450 nm in different solvents at 298 K (2 × 10^{-5} M).

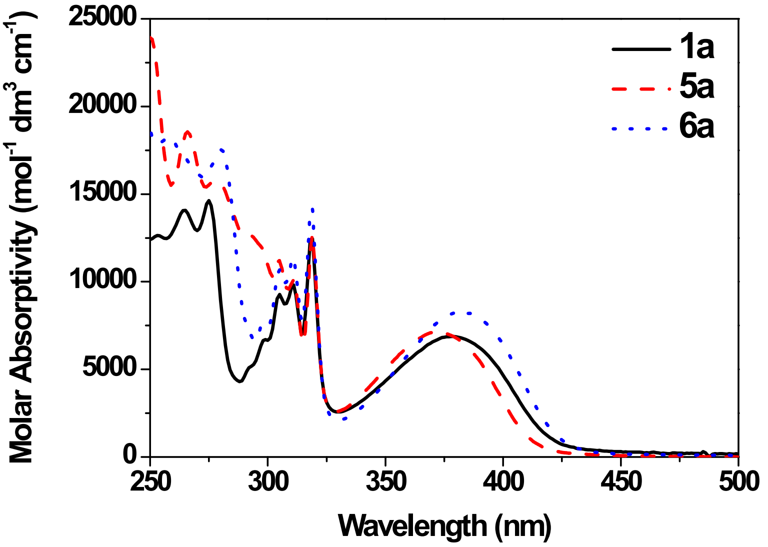

Figure S6 UV-vis absorption spectra of **1a**, **5a** and **6a** in CH_2Cl_2 at 298 K (2×10^{-5} M).

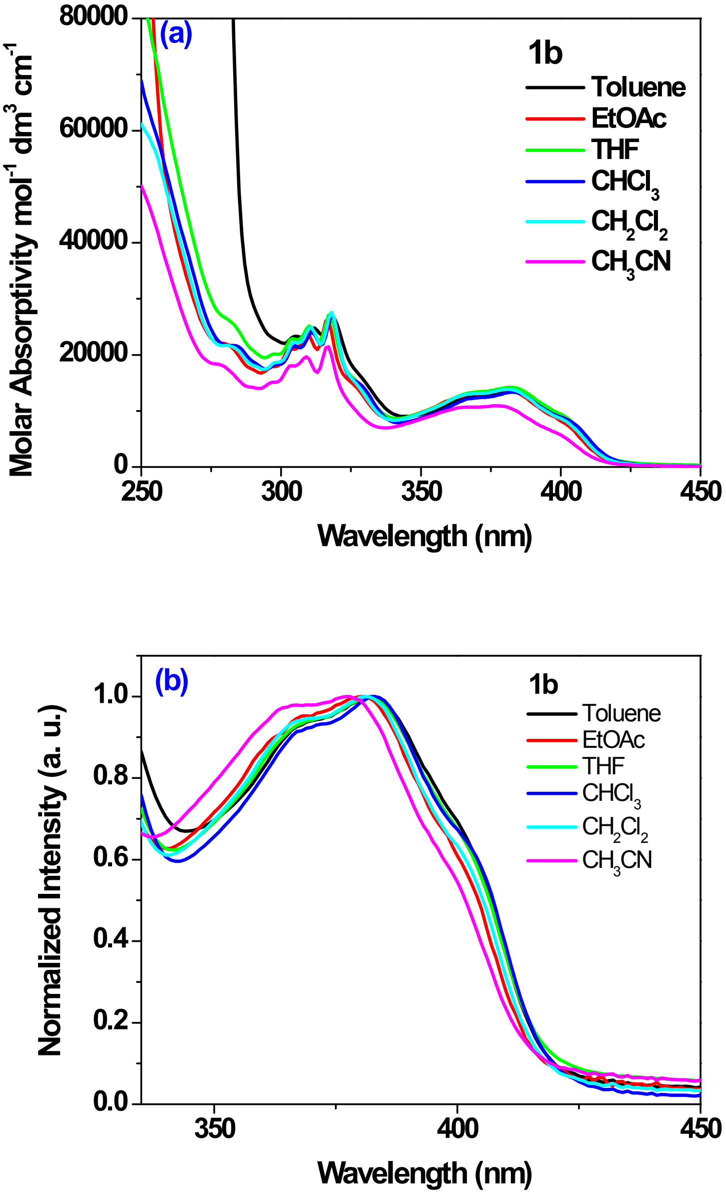

Figure S7 (a) UV-vis absorption spectra and (b) normalized UV-vis absorption spectra of **1b** at 335–450 nm in different solvents at 298 K (2×10^{-5} M).

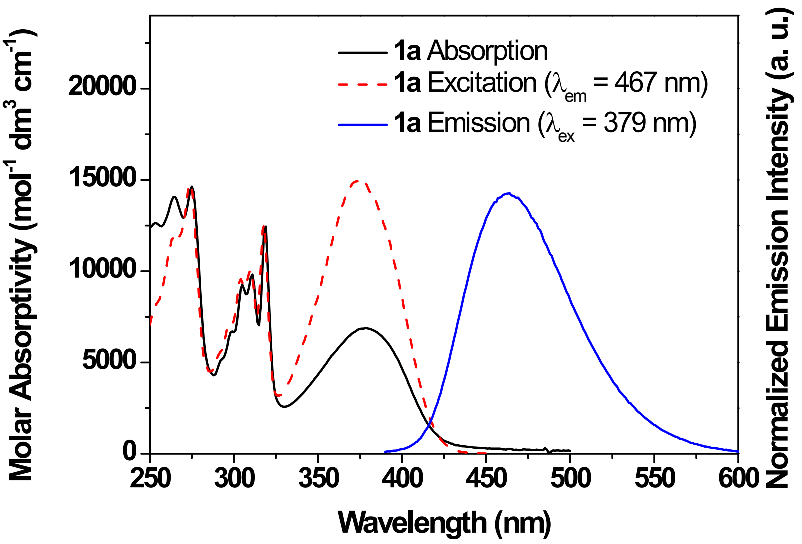
1a Absorption
1a Excitation (λem = 467 nm)
1a Emission (λex = 379 nm)
Molar Absorptivity (mol-1 dm3 cm-1)
Normalized Emission Intensity (a. u.)
Wavelength (nm)

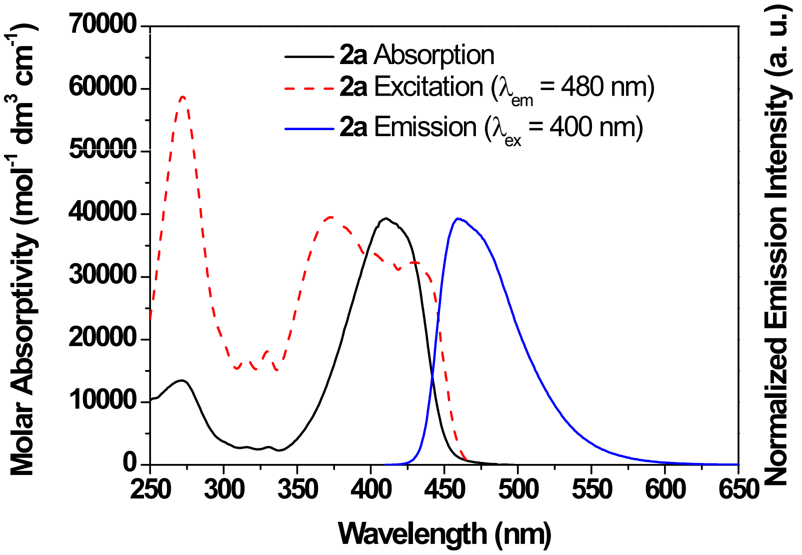
2a Absorption
2a Excitation (λem = 480 nm)
2a Emission (λex = 400 nm)
Molar Absorptivity (mol-1 dm3 cm-1)
Normalized Emission Intensity (a. u.)
Wavelength (nm)

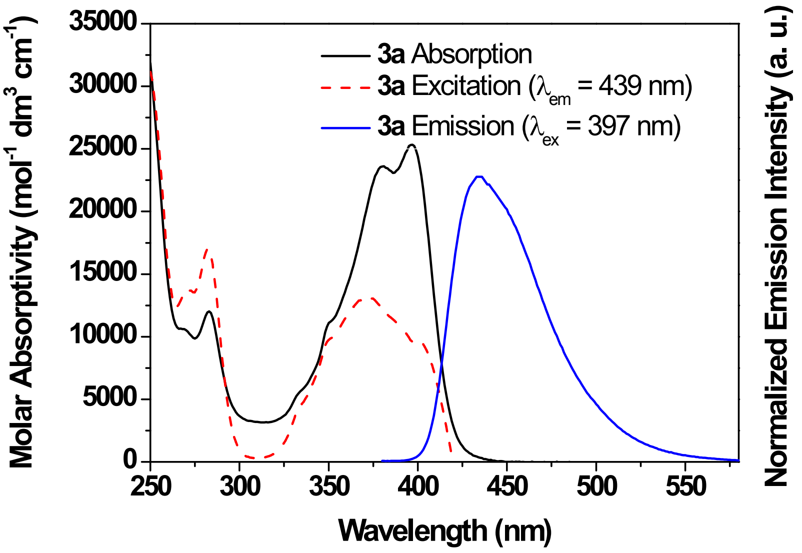
3a Absorption
3a Excitation (λem = 439 nm)
3a Emission (λex = 397 nm)
Molar Absorptivity (mol-1 dm3 cm-1)
Normalized Emission Intensity (a. u.)
Wavelength (nm)

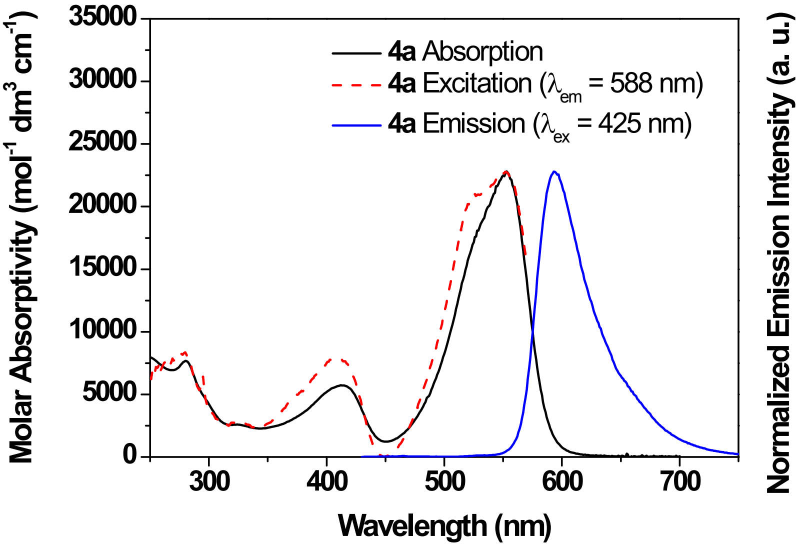

Figure S8 UV-vis absorption (solid line), emission and excitation (dash line) spectra of **1a**–**4a** in degassed CH_2Cl_2 at 298 K.

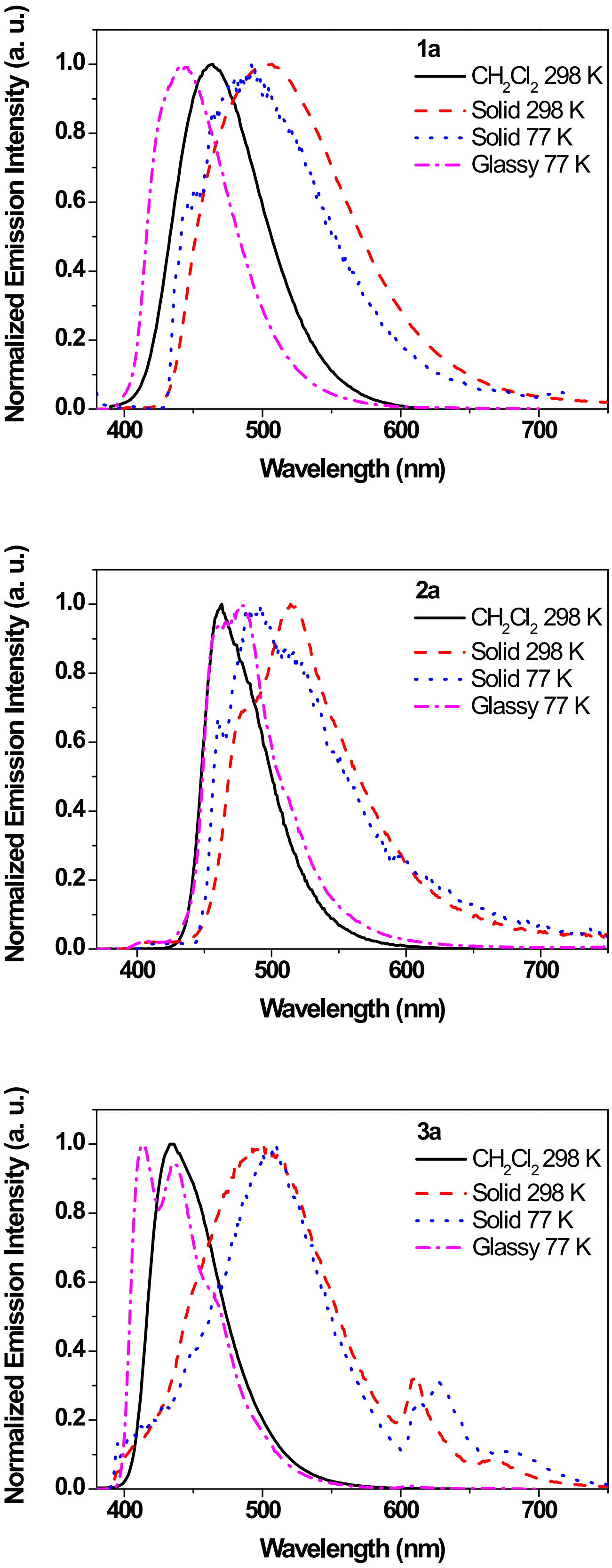

Figure S9 Emission spectra of **1a–3a** in degassed CH_2Cl_2 (2×10^{-5} M), 77 K glassy solution and in solid state at 298 K and 77 K.

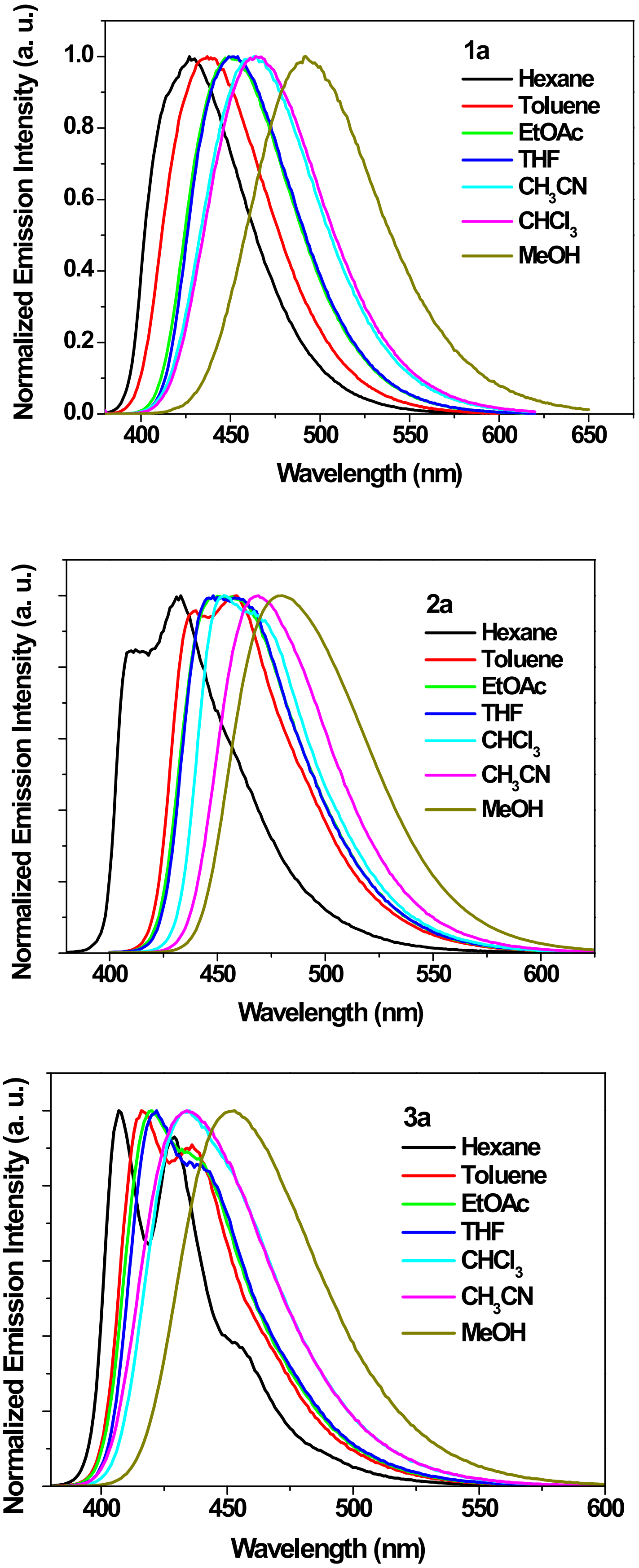

Figure S10 Normalized emission spectra of **1a**–**3a** in different solvents. (2×10^{-5} M)

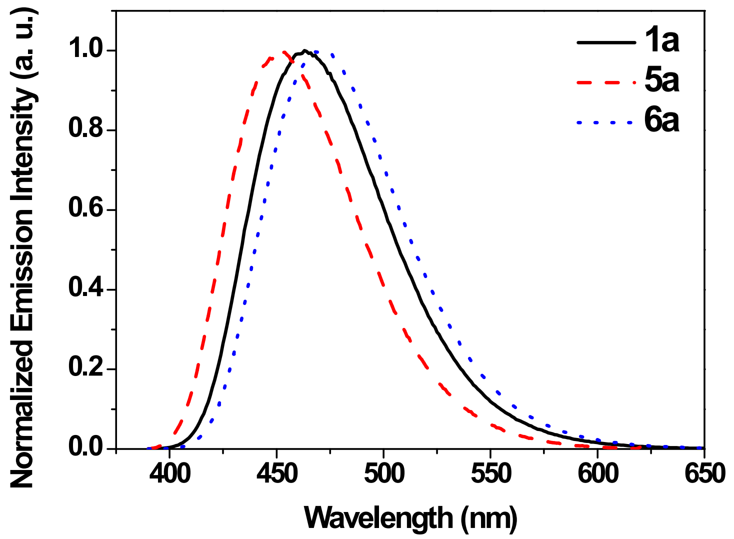

Figure S11 Emission spectra of **1a**, **5a** and **6a** in CH_2Cl_2 at 298 K.

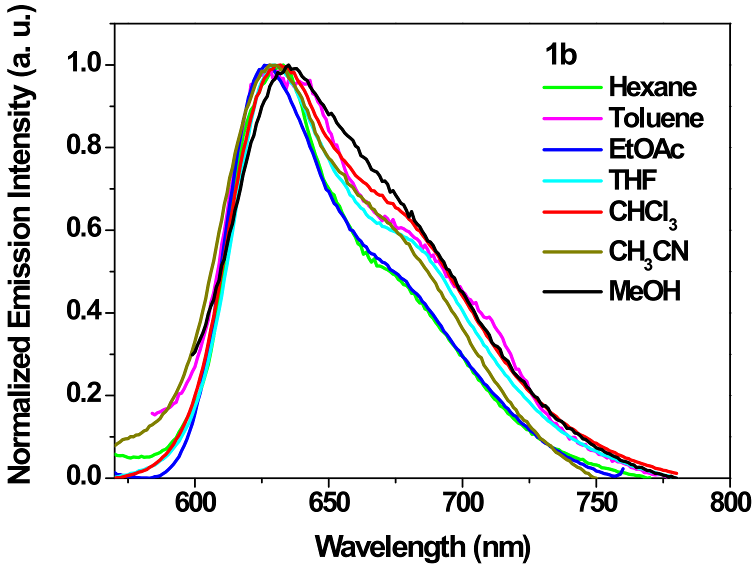

Figure S12 Normalized emission spectra of **1b** in different solvents. (λ_{exc} = 390 nm; 2 × 10^{-5} M)

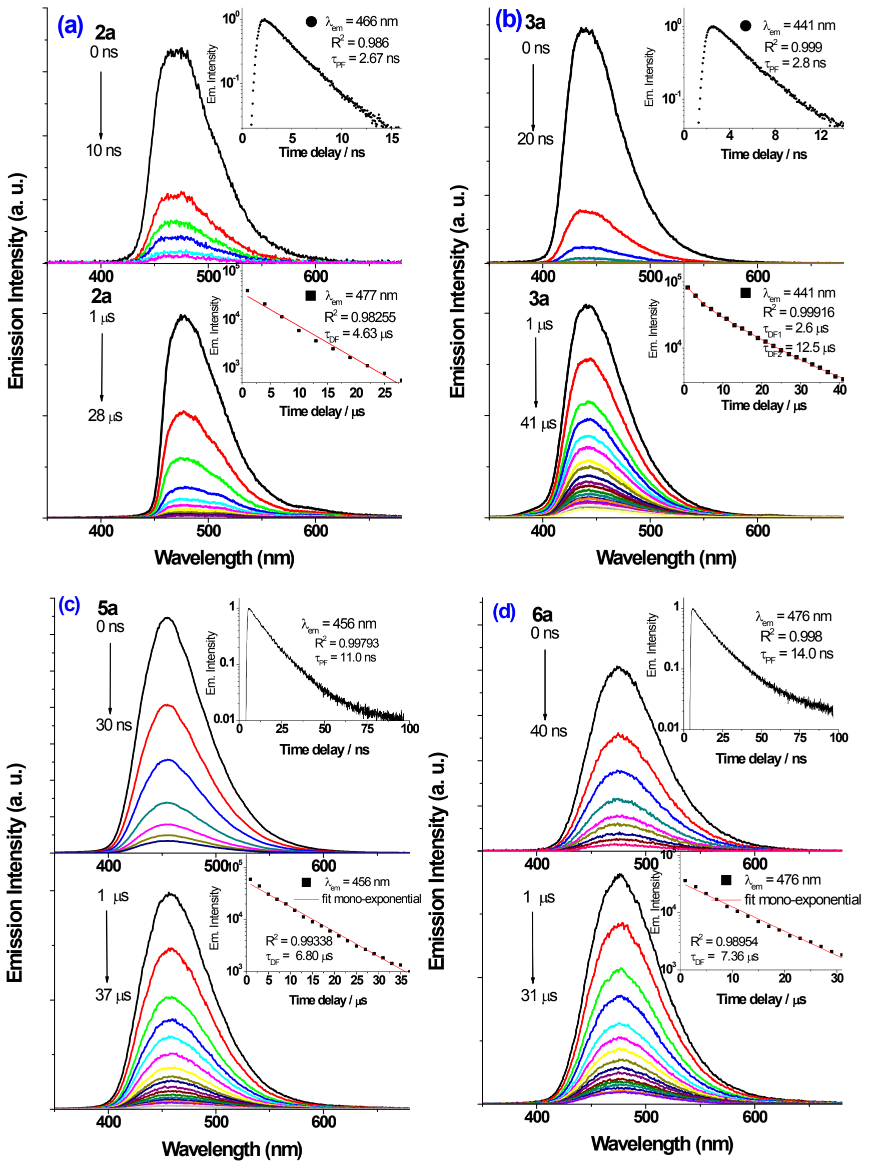

Figure S13 ns-TRE spectra of (a) **2a**, (b) **3a**, (c) **5a** and (d) **6a** recorded from 0–40 ns (top) and after a time delay of 1 μs (bottom) in degassed CH_2Cl_2 (5×10^{-5} M) at 298 K. Insets show the emission kinetic decay traces.

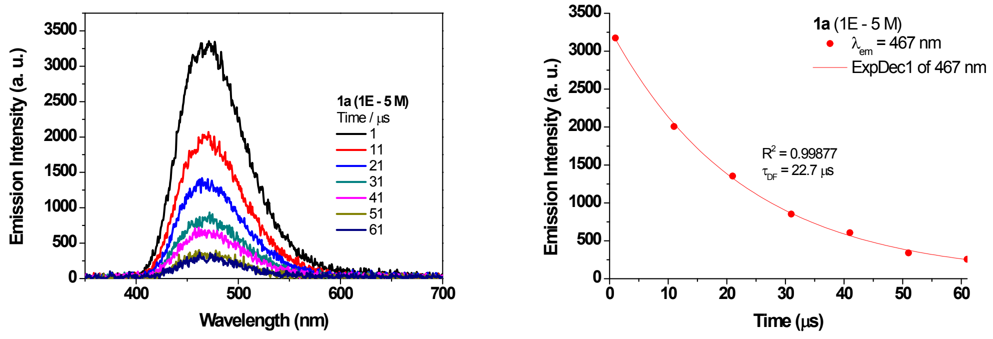

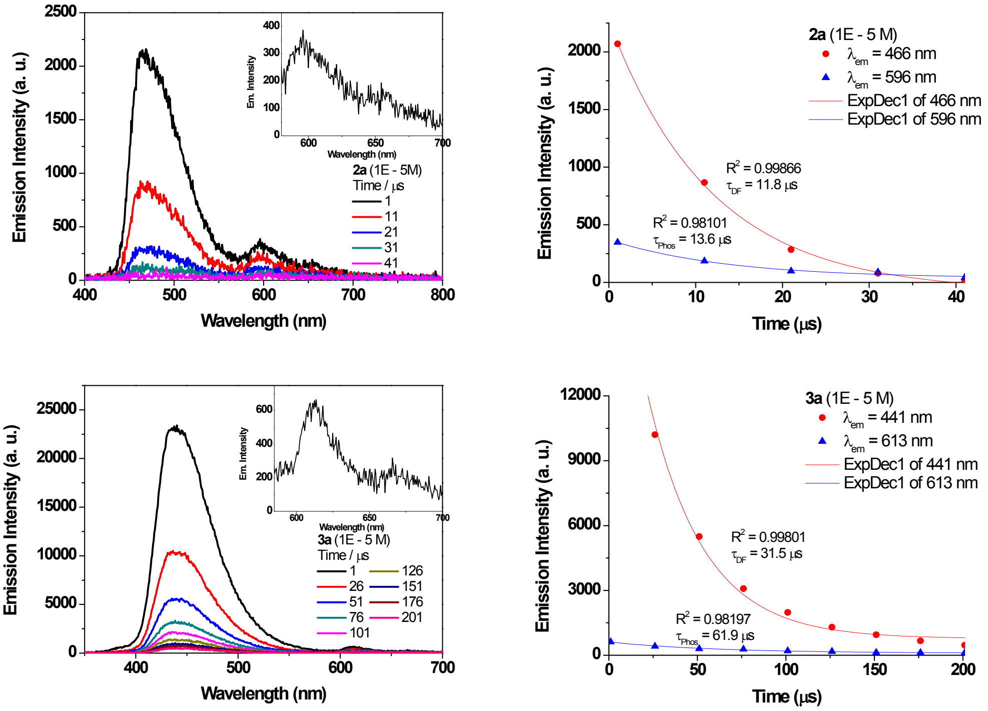

Figure S14 (Left) Time-resolved emission spectra of **1a–3a** in degassed CH_2Cl_2 at 298 K (1×10^{-5} M) recorded at different time intervals. Integration time: 10, 10 and 25 μs for **1a**, **2a**, and **3a** respectively. Insets of **2a–3a** show the magnified emission spectra from 580–700 nm; (Right) the kinetic decay traces at the specified wavelengths; decay time constants were obtained as mono-exponential fit.

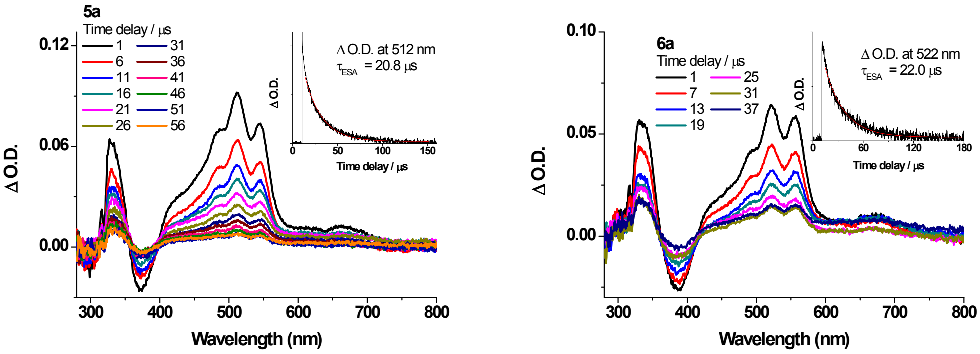

Figure S15 Nanosecond transient absorption (ns-TA) spectra of **5a–6a** recorded at selected decay time in degassed CH_2Cl_2 (5×10^{-5} M) at 298 K; insets show the ESA kinetic trace at the specified wavelength; decay lifetimes were fitted as mono-exponential decay for **5a** and **6a**.

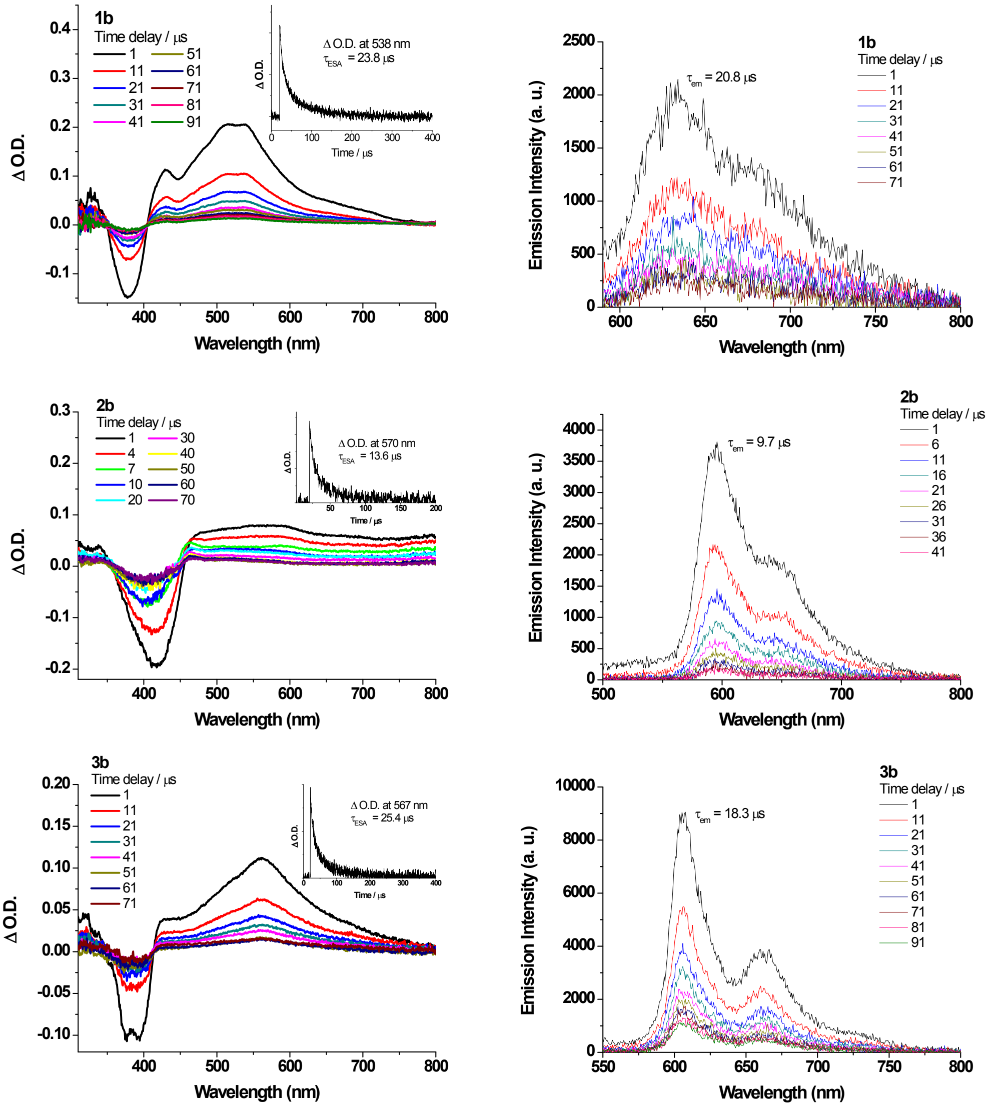

Figure S16 (Left) ns-TA difference spectra and (right) ns-TRE spectra of **1b**–**3b** recorded at selected decay time in degassed CH_2Cl_2 (5×10^{-5} M) at 298 K; insets show the kinetic traces and decay lifetimes of the lowest energy ESA in the spectral region investigated.

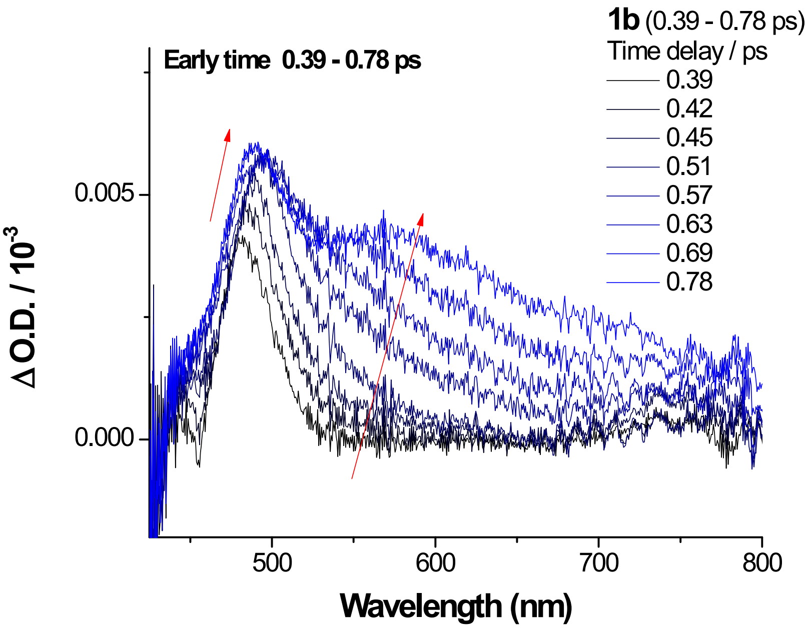

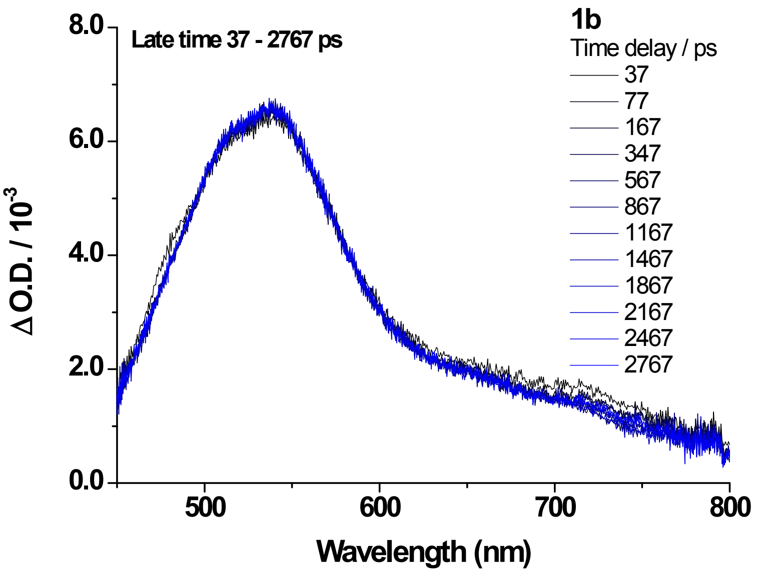

Figure S17 fs-TA difference spectra of **1b** at early (top) and late picosecond time (bottom) recorded in CH_2Cl_2 (5×10^{-5} M) at 298 K.

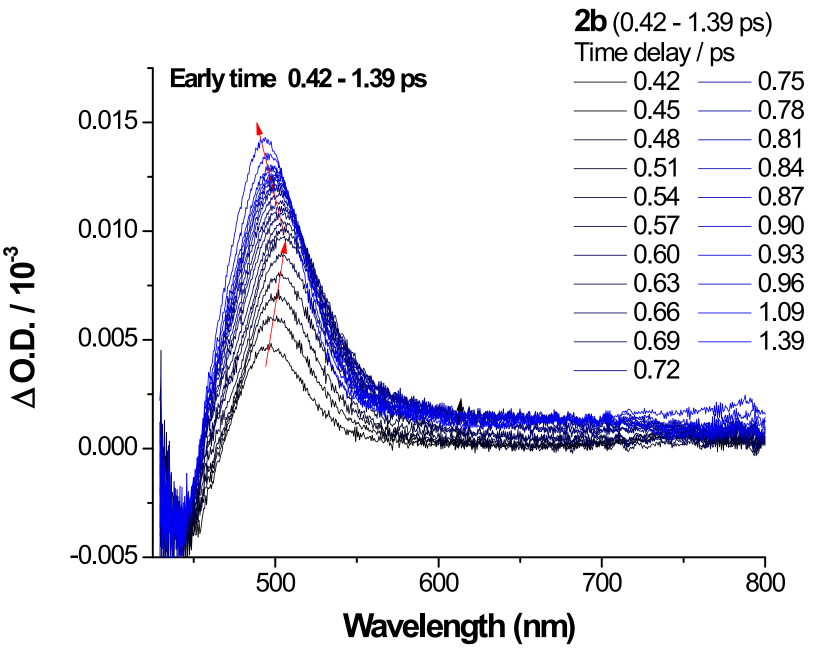

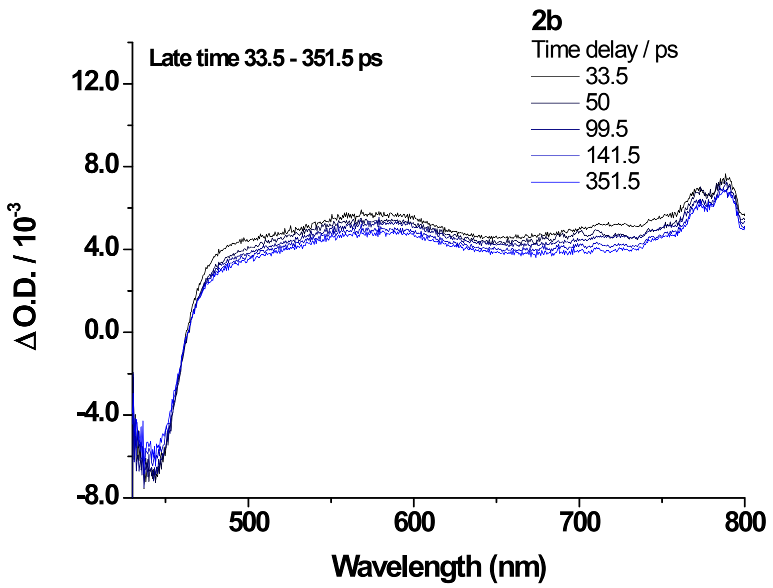

Figure S18 fs-TA difference spectra of **2b** at early (top) and late picosecond time (bottom) recorded in CH_2Cl_2 (5×10^{-5} M) at 298 K.

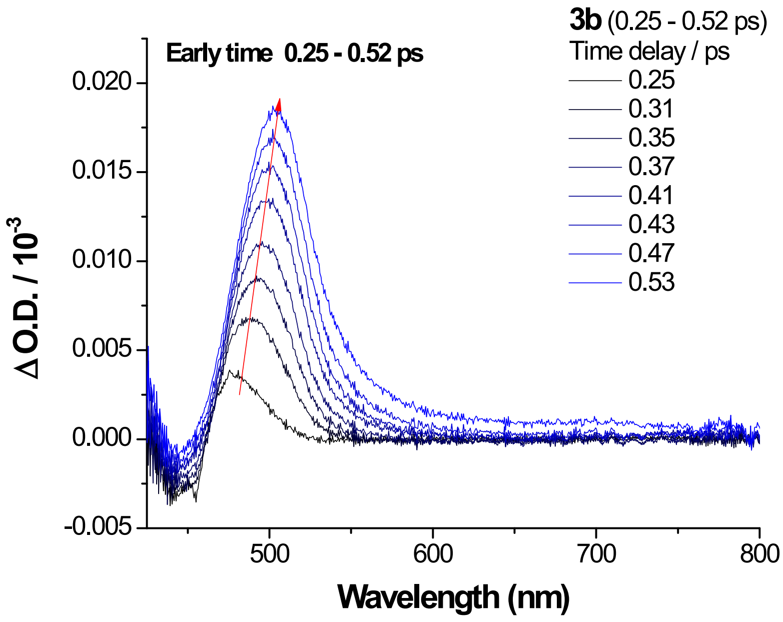

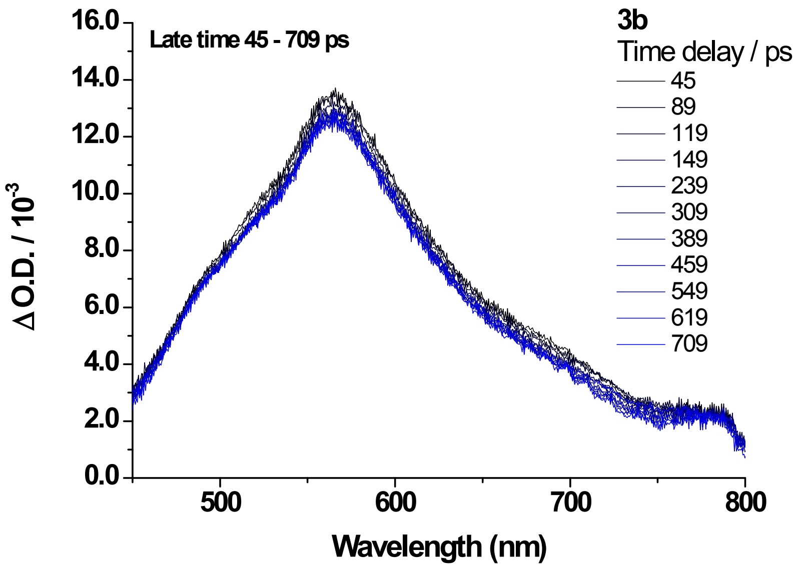

Figure S19 fs-TA difference spectra of **3b** at early (top) and late picosecond time (bottom) recorded in CH_2Cl_2 (5×10^{-5} M) at 298 K.

3. Computational Details

In this work, the hybrid density functional, PBE0,[4] was employed for all calculations using the program package G09.[5] The 6-31G* basis set[6] is used for all atoms except Au, which is described by the Stuttgart relativistic pseudopotential and its accompanying basis set (ECP60MWB).[7] Solvent effect was also included by means of the polarizable continuum model (PCM).[8] Geometry optimizations of the singlet ground state (S_0) were carried out using the density functional theory without symmetry constraints. Frequency calculations were performed on the optimized structures to ensure that they are minimum energy structures by the absence of imaginary frequency (i.e. NImag = 0). Stability calculations were also performed for all the optimized structures to ensure that all the wavefunctions obtained were stable. Vertical excitation energies of the singlet and triplet excited states were calculated based on the time-dependent density functional theory (TDDFT) using the linear response approximation (LR-PCM). Spin-orbit coupling matrix elements between the singlet and triplet excited states are computed as previously reported.[9]

Table S5 Singlet and triplet excited state energies and compositions at the optimized S_0 geometry of **1a** computed by TD-PBE0.

No.	E [cm^{-1}]	λ [nm]	*f*	Major Contribution	Minor Contribution
T1	14642	683	0	HOMO→LUMO (89%)	H–4→LUMO (7%)
S1	25237	396	0.2736	HOMO→LUMO (99%)	
T2	25811	387	0	H–3→LUMO (89%)	HOMO→L+1 (6%)
T3	27681	361	0	H–4→LUMO (43%), HOMO→L+1 (28%)	H–3→LUMO (7%), HOMO→L+4 (5%), H–10→LUMO (5%)
T4	28417	352	0	H–1→LUMO (90%)	H–8→LUMO (3%), H–1→L+1 (2%)
S2	29464	339	0	H–1→LUMO (97%)	H–1→L+1 (2%)

Table S6 Singlet and triplet excited state energies and compositions at the optimized S_0 geometry of **1b** computed by TD-PBE0.

No.	E [cm^{-1}]	λ [nm]	*f*	Major Contribution	Minor Contribution
T1	14980	668	0	HOMO→LUMO (81%)	HOMO→L+1 (8%), H–8→LUMO (5%)
T2	21913	456	0	H–1→LUMO (28%), H–1→L+1 (56%)	H–4→L+2 (4%)
T3	22158	451	0	H–4→L+1 (14%), H–1→L+2 (60%)	H–4→LUMO (5%), H–3→L+2 (5%)
T4	25591	391	0	H–6→LUMO (62%), H–6→L+1 (11%), HOMO→L+1 (11%)	HOMO→L+3 (4%)
S1	25653	390	0.3676	HOMO→LUMO (97%)	

T5	26044	384	0	H–2→LUMO (79%), H–2→L+1 (12%)	
S2	26671	375	0.0395	H–1→LUMO (57%), H–1→L+1 (41%)	
T6	26725	374	0	H–8→LUMO (14%), H–6→LUMO (18%), HOMO→L+1 (40%)	HOMO→LUMO (6%), HOMO→L+3 (5%)
S3	26971	371	0.003	H–2→LUMO (89%)	H–2→L+1 (6%)

Table S7 Singlet and triplet excited state energies and compositions at the optimized S_0 geometry of **4a** computed by TD-PBE0.

No.	E [cm^{-1}]	λ [nm]	*f*	Major Contribution	Minor Contribution
T1	12005	833	0	HOMO→LUMO (87%), H–1→LUMO (13%)	
T2	18071	553	0	H–1→LUMO (79%), HOMO→LUMO (12%)	
S1	20182	495	0.47	HOMO→LUMO (96%)	H–1→LUMO (4%)
T3	23137	432	0	H–6→LUMO (16%), H–4→LUMO (38%), H–3→LUMO (24%)	H–8→LUMO (4%), H–5→LUMO (8%)
T4	23593	424	0	H–6→LUMO (11%), H–4→LUMO (17%), H–3→LUMO (54%)	H–11→LUMO (3%), H–5→LUMO (3%), H–3→L+3 (3%)
T5	26511	377	0	H–2→LUMO (98%)	
S2	26900	372	0.1485	H–2→LUMO (65%), H–1→LUMO (33%)	

S3	26958	371	0.2934	H–2→LUMO (34%), H–1→LUMO (62%)	HOMO→LUMO (2%)

Table S8 Singlet and triplet excited state energies and compositions at the optimized S_0 geometry of **4b** computed by TD–PBE0.

No.	E [cm^{-1}]	λ [nm]	*f*	Major Contribution	Minor Contribution
T1	12344	810	0	HOMO→LUMO (87%) H–3→LUMO (13%)	
T2	18717	534	0	H–3→LUMO (76%) HOMO→LUMO (13%)	
S1	20821	480	0.5847	HOMO→LUMO (95%)	H–3→LUMO (4%)
T3	22013	454	0	H–1→L+1 (79%)	H–5→L+2 (5%), H–1→LUMO (5%)
T4	22153	451	0	H–5→L+1 (18%), H–1→L+2 (60%)	H–4→L+2 (5%)
T5	23036	434	0	H–9→LUMO (26%), H–8→LUMO (34%), H–6→LUMO (25%)	H–11→LUMO (4%)
T6	23505	425	0	H–9→LUMO (16%), H–8→LUMO (13%), H–6→LUMO (51%)	H–7→LUMO (4%), H–3→LUMO (3%), H–6→L+5 (3%)
T7	24985	400	0	H–2→LUMO (92%)	H–10→LUMO (3%), H–2→L+1 (3%)
S2	25290	395	0.0001	H–2→LUMO (95%)	H–10→LUMO (3%)
T8	25407	394	0	HOMO→L+1 (81%)	H–3→L+1 (5%)
S3	25781	388	0.0064	H–1→LUMO (90%),	

H–1→L+1 (10%)

Table S9 Selected frontier MO surfaces of **1a** and **1b** at the optimized S_0 geometries.

**1a**	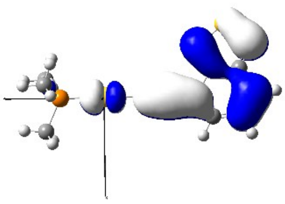 H–4	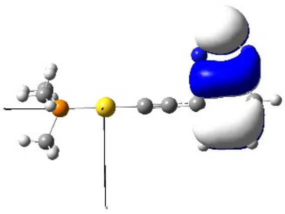 H–3	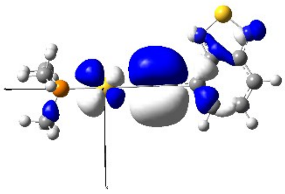 H–1
	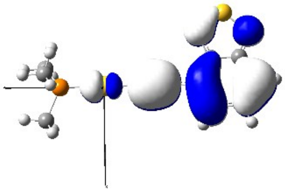 HOMO	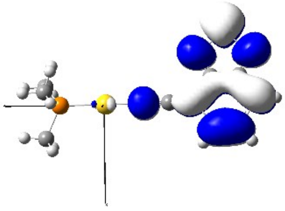 LUMO	
**1b**	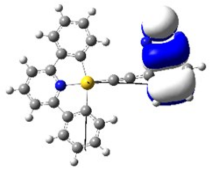 H–6	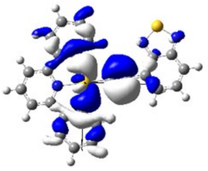 H–2	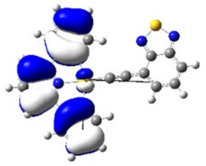 H–1
	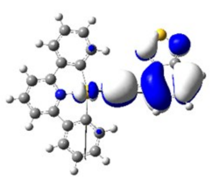 HOMO	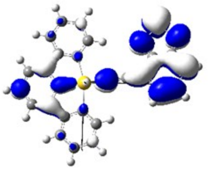 LUMO	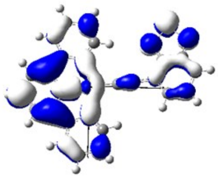 L+1

Table S10 Selected frontier MO surfaces of **4a** and **4b** at the optimized S_0 geometries.

**4a**	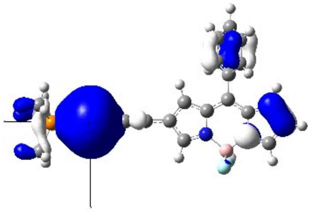 H–5	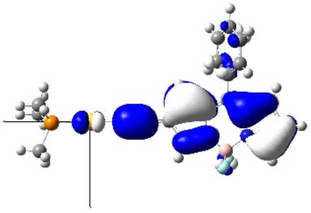 H–1	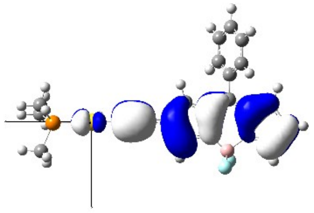 HOMO
	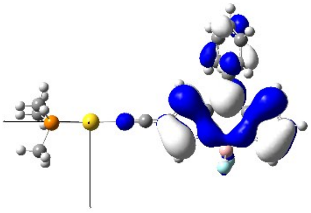 LUMO		
**4b**	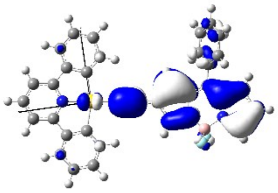 H–3	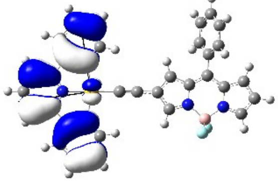 H–1	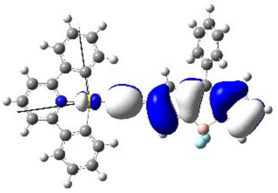 HOMO
	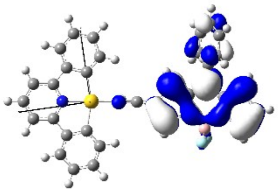 LUMO	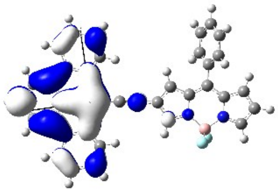 L+1	

4. References

[1] J. N. Demas, G. A. Crosby, *J. Phys. Chem.*, 1971, **75**, 991.

[2] L. Du, R. Zhu, J. Xue, Y. Du and D. L. Phillips, *J. Raman Spect.*, 2015, **46**, 117.

[3] K. Pilgram, M. Zupan and R. Skiles, *J. Heterocyclic Chem.,* 1970, **7**, 629.

[4] C. Adamo and V. Barone, *J. Chem. Phys.,* 1999, **110**, 6158.

[5] M. J. Frisch, *et al.*, *Gaussian 09 (Revision C.01),* Gaussian, Inc., Wallingford CT, 2009.

[6] (a) M. M. Francl, W. J. Petro, W. J. Hehre, J. S. Binkley, M. S. Gordon, D. J. DeFree and J. A. Pople, *J. Chem. Phys.,* 1982, **77**, 3654; (b) P. C. Hariharan and J. A. Pople, *Theor. Chim. Acta,* 1973, **28**, 123.

[7] (a) D. Andrae, U. Haeussermann, M. Dolg, H. Stoll and H. Preuss, *Theor. Chim. Acta,* 1990, **77**, 123; (b) J. M. L. Martin and A. Sundermann, *J. Chem. Phys.,* 2001, **114**, 3408.

[8] M. Cossi, G. Scalmani, N. Rega and V. Barone, *J. Chem. Phys.,* 2002, **117**, 43.

[9] G. S. M. Tong, K. T. Chan, X. Chang and C.-M. Che, *Chem. Sci.*, 2015, **6**, 3026.
